# Quality Assurance of Health Wearables Data: Participatory Workshop on Barriers, Solutions, and Expectations

**DOI:** 10.2196/15329

**Published:** 2020-01-22

**Authors:** Robab Abdolkhani, Kathleen Gray, Ann Borda, Ruth DeSouza

**Affiliations:** 1 Health and Biomedical Informatics Centre The University of Melbourne Melbourne, Victoria Australia

**Keywords:** remote sensing technology, data quality assurance, patient-generated health data, wearable devices, participatory research

## Abstract

**Background:**

The ubiquity of health wearables and the consequent production of patient-generated health data (PGHD) are rapidly escalating. However, the utilization of PGHD in routine clinical practices is still low because of data quality issues. There is no agreed approach to PGHD quality assurance; therefore, realizing the promise of PGHD requires in-depth discussion among diverse stakeholders to identify the data quality assurance challenges they face and understand their needs for PGHD quality assurance.

**Objective:**

This paper reports findings from a workshop aimed to explore stakeholders’ data quality challenges, identify their needs and expectations, and offer practical solutions.

**Methods:**

A qualitative multi-stakeholder workshop was conducted as a half-day event on the campus of an Australian University located in a major health care precinct, namely the Melbourne Parkville Precinct. The 18 participants had experience of PGHD use in clinical care, including people who identified as health care consumers, clinical care providers, wearables suppliers, and health information specialists. Data collection was done by facilitators capturing written notes of the proceedings as attendees engaged in participatory design activities in written and oral formats, using a range of whole-group and small-group interactive methods. The collected data were analyzed thematically, using deductive and inductive coding.

**Results:**

The participants’ discussions revealed a range of technical, behavioral, operational, and organizational challenges surrounding PGHD, from the time when data are collected by patients to the time data are used by health care providers for clinical decision making. PGHD stakeholders found consensus on training and engagement needs, continuous collaboration among stakeholders, and development of technical and policy standards to assure PGHD quality.

**Conclusions:**

Assuring PGHD quality is a complex process that requires the contribution of all PGHD stakeholders. The variety and depth of inputs in our workshop highlighted the importance of co-designing guidance for PGHD quality guidance.

## Introduction

The health care industry is rapidly moving toward patient-centered care, in which patients actively contribute to their health care [1]. An example of a patient-centered care model, remote patient monitoring keeps patients outside the clinical setting while monitoring their health status. In these interventions, patients actively engaged in their health care and played an increasingly important role in sharing responsibilities with health care providers [2].

### Background

Health wearables are a key component of remote patient monitoring. These devices have the capability to continuously collect, process, and display health data automatically in real time, which may improve patients’ awareness of their health outside the clinical setting and enhance self-management [3].

Health wearables in the market are of 2 types: medical grade and consumer wearables. Medical wearables are designed to collect data relevant to specific health conditions, such as continuous glucose monitoring (CGM), to monitor blood glucose levels in people with diabetes. However, consumer wearables are often designed to collect general wellness data, such as fitness trackers that are used by patients to be aware of their physical activity level, sleep quality, mood, and heart rate. Some types of wearables connect to an associated mobile app to display real-time processed data, as well as to a patient portal where both patients and health care providers access passive data. Depending on the wearable platform, patients may also be required to manually enter other data types into the mobile app, in addition to the automatic data captured by the sensors in the wearable device.

Data produced by wearables are usually called patient-generated health data (PGHD), as the data collection relies on patients’ (rather than clinicians’) control and occurs outside the clinical environment [4]—a highly complex process. Large volumes of data collected from disparate wearables on an ongoing basis and under various situations bring possibilities for PGHD to be impacted by various technical, behavioral, operational, and organizational issues at the time of storage, transmission, analysis, or access for shared decision making that can lead to poor quality data. For example, wearable dysfunctionality [5-8], inaccurate calibration [6], incorrect manual inputs [5-9], complex data visualization [6,10], or not collecting data for a period of time [9-12] result in lack of trust in PGHD for clinical decision making. During the PGHD transmission stage, patients may share their data through a home network, their mobile wireless network, or a public wireless network, all of which might affect data transmission [5]. In addition, some of the platforms used during PGHD flow operate outside health information systems and may not be trusted because of concerns about their security and data privacy [6,9]. It is still challenging to define and implement solutions for integrating existing data in electronic medical records (EMRs) with PGHD from patients’ own wearables and develop widely accepted interoperability standards for PGHD exchange [6-11]. Moreover, concerns exist regarding consumer wearables, as they are not yet regulated, and there is uncertainty about their promise in achieving the quality required in optimal PGHD collection [12,13]. Regarding human factors, an individual’s intention to use wearables for general wellness tracking or for monitoring a specific health condition can strongly affect the quality of PGHD that these devices collect [8,13-15].

Health care providers may face large volumes of inaccurate, incomplete, irrelevant, and nonunderstandable PGHD, without solid principles for dealing with these data. The result is concerns about reliability and no confidence in using PGHD in routine clinical practice [16,17].

These issues of PGHD quality make it difficult to understand PGHD’s usefulness for health care, and these hinder adoption of these data as a systematic part of clinical practices [18]. Owing to this, remote patient monitoring is often initiated on a small scale for a short time and focuses on a single disease. A 2018 survey of over 20,000 health care consumers in 28 countries revealed nonsignificant adoption of PGHD [19].

There is little evidence in the research and industry literature that the quality of PGHD from wearables is sufficiently well managed to enable them to be trusted as health data or that such data can be used safely and effectively in clinical care. Furthermore, health care organizations lack awareness about PGHD quality from wearable devices [13,20].

### Objectives

Assuring PGHD quality requires efforts not only from health care providers but also from patients, their caregivers, and health consumers in general. Consumers, for example, have different needs from health care providers; therefore, their attitudes toward PGHD will differ, as well as the impact on data quality [21]. However, PGHD stakeholders are not limited to consumers and providers; other stakeholders, such as wearable manufacturers, contribute to PGHD transmission from outside the clinical setting, and their activities may similarly impact PGHD quality [16].

Therefore, it is critical to enhance general awareness of the quality of PGHD from wearables.

The objective of this study was to identify current challenges stakeholders face with various attributes of PGHD quality, define potential solutions to overcome those challenges, and facilitate a process where they could workshop their data quality needs and expectations with other stakeholders.

## Methods

### Study Design

We conducted a half-day workshop in May 2019 on the campus of an Australian University located in a major health care precinct. This was part of a larger project using a range of inputs to develop PGHD quality assurance guidance. The workshop received human research ethics approval from the University of Melbourne (Ethics ID: 18532521).

### Participant Recruitment

Participants were eligible to attend the workshop, who were at least 18 years old and had experience of PGHD use in clinical care, including people who identified as health care consumers, clinical care providers, wearables suppliers, health information specialists, PGHD integration service providers, data analysts, and health service managers. Expressions of interest were sought via an open call and a Web-based registration form distributed via internal and public news and media channels, as well as professional organizations for digital health in Australia. At the point of recruitment or before, as needed, gaps were filled with personal invitations and via snowball sampling, for example, via key contacts in specific organizations where the researchers had existing relationships.

### Participants

In total, 18 participants took part in the workshop. The participants included 8 health consumers and consumer advocates, 5 clinicians, 3 health information professionals, and 2 wearable and data integration company representatives.

### Workshop Structure

#### On-Site Registration

Each participant was given a package of stationery materials, the consent form, the Plain Language Statement, and a copy of the presentation slides.

#### Researcher’s Presentation

Participants were informed about the workshop purpose and its structure. In addition, findings from previous parts of the project [6,14] were elucidated. The concept of PGHD quality and its 7 aspects—accessibility, accuracy, completeness, consistency, interpretability, relevancy, and timeliness—adapted from a comprehensive data quality framework developed for clinical data quality assurance by the Australian Capital Territory [22] were explained through scenarios (Multimedia Appendix 1) to enable the participants to identify the various circumstances that might impact each PGHD quality aspect and identify the data management stage in which the quality aspect might require consideration. We used CGM devices as a medical wearable example, and we used fitness trackers as a consumer wearable example in this workshop.

#### Group Discussion 1

**Figure 1:**
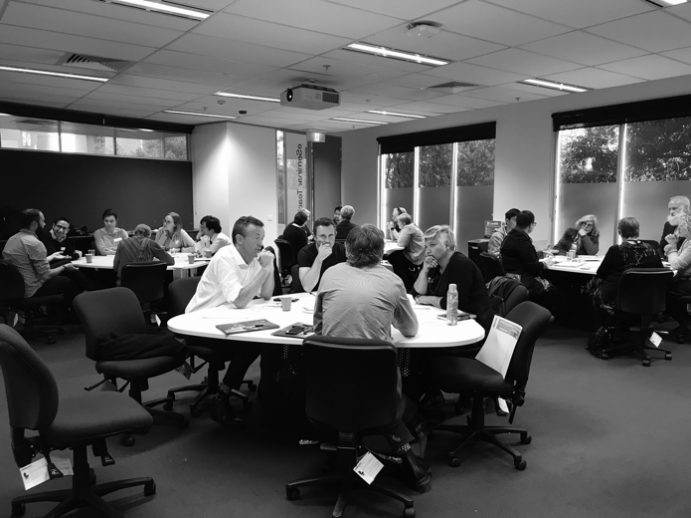
Participants’ discussions in 4 groups of patient-generated health data stakeholders.

**Figure 2:**
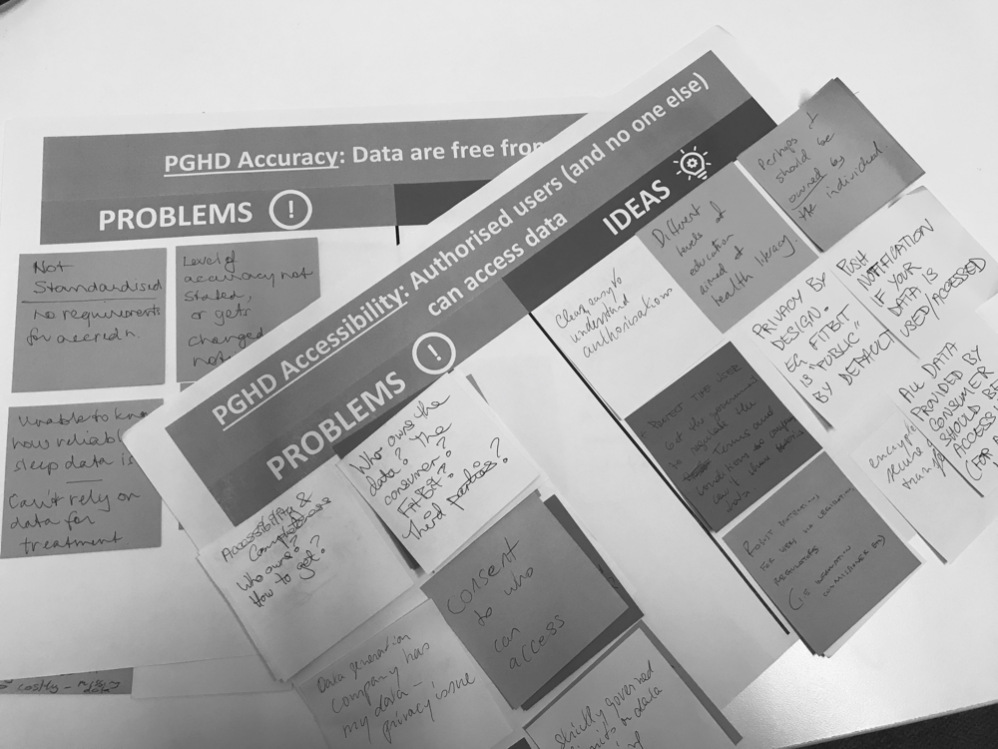
Worksheets of patient-generated health data quality problems and ideas.

The facilitators directed participants into 4 groups, each with a cross section of different stakeholders (Figure 1). Groups discussed problems related to PGHD quality. Participants contributed verbally, wrote ideas on sticky notes, and placed these on their group worksheet (Figure 2). A total of 1 facilitator managed each group to direct discussions and assist in timekeeping. Small group discussions ran for 30 min. Thereafter, in a 15-min session, each facilitator took turns presenting to the whole room the consolidated deliberations of the facilitator’s small group. The facilitators wrote the key findings on whiteboards to be available during the break.

#### Break

Participants walked around the workshop room to view the discussion findings on whiteboards and exchange further ideas with participants, in preparation for the next part.

#### Group Discussion 2

**Figure 3:**
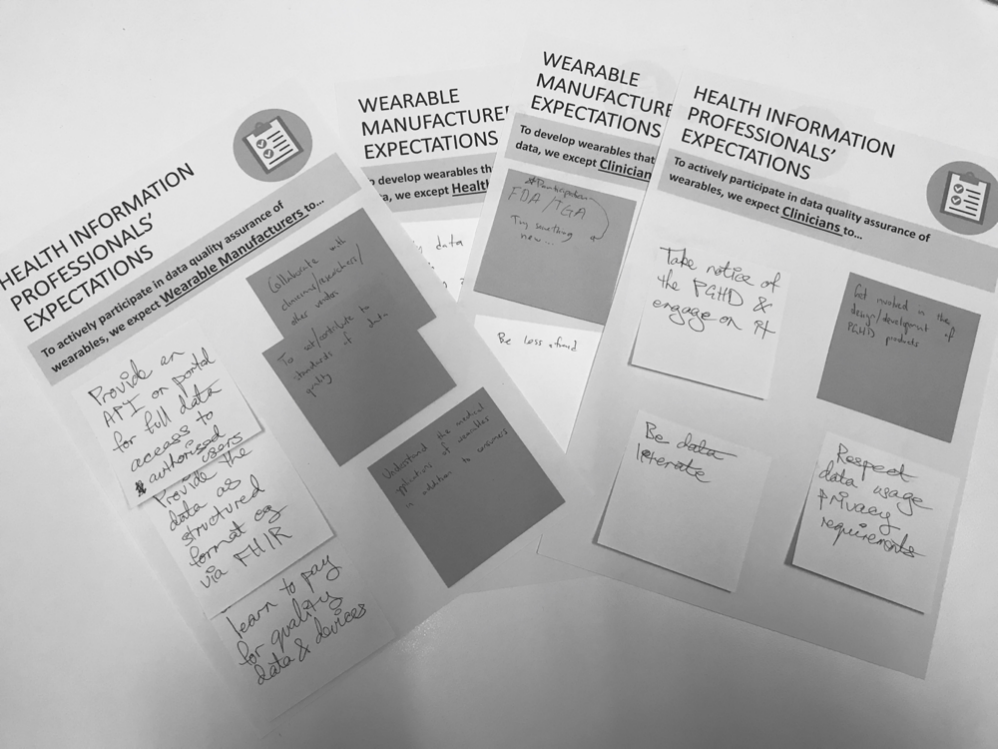
Worksheets of patient-generated health data stakeholder’s expectations.

The facilitators directed participants into 4 differently configured groups. Each group took on a *role* of a key stakeholder—health care consumer, health care provider, health information professional, or wearables manufacturer. Groups discussed and translated their ideas into a set of their expectations of other stakeholder groups, on worksheets (Figure 3). This task took 30 min, followed by 15 min in which each facilitator set out and spoke to the respective group’s expectations on whiteboards.

#### Conclusion

The researchers pointed out the outcomes and future work implications. Participants were informed about the next steps of the research project and how they may stay engaged if they are interested.

### Data Collection and Analysis

Data collection was done by facilitators capturing written notes of the proceedings as attendees engaged in participatory design activities in written and oral formats, using a range of whole-group and small-group interactive methods. The group facilitators’ summing up presentations on boards and sticky notes were photographed. Deductive analysis based on 7 PGHD quality aspects (accessibility, accuracy, completeness, consistency, interpretability, relevancy, and timeliness) and inductive thematic content analysis were used as the primary data analysis methods. The initial data analysis was conducted by the PhD researcher, which was then reviewed by 3 supervisors. Thereafter, all of the 4 researchers discussed the results together in several meetings until consensus was achieved.

## Results

The workshop findings are organized thematically as problems and solutions related to each health data quality dimension, as well as stakeholders’ expectations of each other, to assure PGHD quality.

### Data Quality Challenges and Solutions

Table 1 illustrates the current problems and potential solutions that mixed-stakeholder small groups raised for each data quality aspect.

**Table 1:** Patient-generated health data quality problems and potential solutions.

Definition	Problems	Potential solutions
Patient-generated health data accessibility (authorized users can access data)	Lack of transparency on who owns the data Lack of consent for continuous data collection and use Lack of health consumers’ access to raw data Data hacking	Develop data ownership principles Design notifications in the wearable platform to alert consumers once data are accessed by others Provide dynamic data authorization Provide access to raw data by health consumers Define wearable cybersecurity standards Create data encryption techniques Consider privacy in the wearable design Develop layered consent for various data from different devices
Accuracy (data are free from errors)	Inaccurate data because of the use of different wearables with different accuracy standard levels Errors in wearable functionality Mistakes in manual data entry Lack of data editing functionalities	Define accurate levels of measurements Wearable manufacturers adopt accuracy-related feedback given by consumers and clinicians Enable data edit functionality in the wearable platforms
Completeness (there are no data missing)	Lack of access to internet to send the collected data Battery problems Incompleteness of data entered manually Lack of data synchronization during change of time zones Incompleteness of data because of the wearable dysfunction Deliberate data omissions	Design notification to provide an alert for missing data Consumers’ education and engagement
Consistency (data from different devices convey the same meaning)	Lack of awareness of data flow and data management Data inconsistency because of using various wearables with different platforms	Develop data consistency checking mechanisms to correlate with other data sources Incorporate data with the clinical workflow
Interpretability (the data presentation highlights the key message)	Presentation of large volumes of data Lack of contextual data from consumer wearables to supplement medical wearables data to be easily understood Data presentations vary among different wearables	Collect contextual data Design standardized data presentation formats for clinicians, despite the variety of wearables used
Relevancy (the data being collected are pertinent to the standard of care)	Different clinical judgement on data relevancy Cyberchondria; overthinking of relevancy of collected data to a specific health condition	Improve health literacy to understand the relevance of data to the standards of care Provide shared understanding of data relevancy among consumers and clinicians
Timeliness (up-to-date data are available when needed)	High volume of unfiltered data to be timely Lack of consensus among patient-generated health data stakeholders about the definition of timeliness depending on the patient’s status (stable or unstable and at risk) Wearable design often determines when data are available	Automation and artificial intelligence to accelerate data filtering so that important data can be available in a timely manner Enable consumers to take responsibility for deciding when health issues need to be escalated Design alerts for critical indicators to patients and clinicians

The findings in Table 1 show the dynamic situations in which patients collect PGHD through wearables and associated components, including mobile apps and portals. Situations are also subject to various technical, environmental, operational, and behavioral factors. These factors largely involve the design and performance of the wearable platforms, the fragmentation in clinical setting infrastructure to utilize PGHD from disparate wearables, and the stakeholders’ literacy levels in their engagement with PGHD collection and use.

The participants offered solutions for each PGHD quality attribute, which can be categorized into 5 overall ideas, namely (1) concerning redesign and development of wearables in a way to allow meeting PGHD quality requirements; (2) creation of clinical and administrative policies to address data quality assurance, such as policies on PGHD ownership, standard measurements, and data management policies; (3) stakeholders’ engagement and education; (4) integration of PGHD with clinical workflows and electronic health record systems, which demands creation of terminologies, data exchange protocols, and data analytics; and (5) defining who in the workforce is responsible for PGHD quality assurance procedures.

### Patient-Generated Health Data Stakeholders’ Expectations

During the second group discussion, participants from each of the 4 stakeholder groups—consumers, clinicians, health information professionals, and wearable manufacturers—stated their expectations of what each of the other PGHD stakeholder groups could do to assure PGHD quality. The groups’ expectations are explained in Tables 2-5. There were no expectations of health information professionals expressed by consumers and wearable manufacturers.

**Table 2:** Consumers’ expectations of other patient-generated health data stakeholder groups.

Consumers’ expectations of PGHD^a^ stakeholders	Details
Clinicians	*Consent:* Consumers expect that clinicians provide informed consent, addressing how consumers and other users can access and use PGHD securely and transparently *Flexibility:* Clinicians are expected to adopt PGHD from new wearable platforms that consumers use and undertake processes to evaluate quality of their data *PGHD collection strategies:* Clinicians can educate consumers on the best practices of PGHD collection and sharing *Collaboration with wearable manufacturers:* Effective collaboration and shared responsibilities among clinicians and wearable manufacturers can lead to clear instructions for consumers in PGHD collection and sharing
Wearable manufacturers	*Code of conduct:* Wearable manufacturers are expected to provide a transparent code of conduct to consumers to identify their rights in using the products *Cost:* Consumers want to access their data from wearable manufacturers for free *Transparency:* Build transparency around the purpose and use of PGHD to enable consumers to have more governance of their data *Person-centered design:* Wearables can be developed on the basis of person-centered care models

^a^PGHD: patient-generated health data.

**Table 3:** Clinicians’ expectations of other patient-generated health data stakeholder groups.

Clinicians’ expectations of PGHD^a^ stakeholders	Details
Consumers	*Trust:* Understanding of PGHD integration into electronic medical records and combination with other clinical data, enhances honesty and enthusiasm among consumers toward the collection of accurate and complete PGHD *Partnership:* Consumers should realize their essential partnership with clinicians in remote patient monitoring. They need to trust their clinicians’ competencies as the first point of decision making for their care
Health information professionals	*Collaborative participation:* Owing to lack of time and burnout on the clinical staff team, clinicians suggest the involvement of health information professionals to analyze and process PGHD. This step should come before PGHD are made available to the clinicians to provide meaningful information for decision making during the clinical consultation *Data governance:* Health information professionals within health care settings should take part in developing PGHD governance strategies and inform clinicians on the best practices for PGHD use *Consumer wearable evaluation:* Health information professionals can assess the contextual data provided by consumer wearables and inform clinicians on PGHD quality from these devices *Technical infrastructure development:* Health information professionals can potentially invest in appropriate information technology infrastructure that enables PGHD integration with electronic medical record systems at scale
Wearable manufacturers	*Dynamic wearable testing:* Need for routine device testing and to collaborate with clinicians to develop strategies for continuous wearable assessment through various clinical studies *Integrity with standards of care:* Wearables should be exclusively designed for the intended purpose of use, and they should align with the standards of care in health care settings

^a^PGHD: patient-generated health data.

**Table 4:** Health information professionals’ expectations of other patient-generated health data stakeholder groups.

Health information professionals’ expectations of PGHD^a^ stakeholders	Details
Consumers	*Consistent data sharing:* Consumers can discuss their preferred wearables and associated platforms with the remote monitoring team to identify ways to share PGHD consistently. *Data sharing authorization:* consumers are expected to authorize their data to be shared.
Clinicians	*PGHD incorporation with other clinical data:* Clinicians can help in incorporating PGHD with other clinical data, as part of the patient record. *Digital health literacy:* Clinicians are expected to undertake training on PGHD and wearable use.
Wearable manufacturers	*Data exchange standards:* Wearable manufacturers should develop devices that comply with defined data exchange standards in health care settings.

^a^PGHD: patient-generated health data.

**Table 5:** Health wearables’ manufacturers’ expectations of other patient-generated health data stakeholder groups.

Health wearables’ manufacturers’ expectations of PGHD^a^ stakeholders	Details
Consumers	*Wearable usability feedback:* Consumers are expected to provide continuous feedback about the technical and operational issues they face in the duration of PGHD use.
Clinicians	*New wearables adoption:* Clinicians should be less afraid to try new technologies and test various wearables via trials. *Collaboration:* Clinicians should expand relationships with wearable manufacturers and be open to conduct research to evaluate the wearables.

^a^PGHD: patient-generated health data.

## Discussion

### Principal Findings

Current health data quality assurance approaches are now disrupted by PGHD challenges. Given the potential value of PGHD in health care decision support, their adoption requires an in-depth understanding of data quality.

The investigation of PGHD quality is in its infancy, and few studies have explored it [6,13,18]. Therefore, our workshop provided the participants with an opportunity to carefully investigate PGHD quality in future remote patient monitoring interventions. It was acknowledged by the participants that the process of thinking about PGHD quality and translating into solutions could enhance collaboration among PGHD stakeholders, within and outside clinical settings.

### Diversity of Patient-Generated Health Data Quality Challenges and Potential Solutions

Mixed-stakeholder participant groups raised divergent problems of PGHD quality. Technical factors related to wearable design and functionality affect most of the aforementioned aspects of data quality at the PGHD collection stage. The participants recognized that a cohesive platform to integrate PGHD with current EMR systems has the potential to collect automatic and manual data entries from various wearables and associated apps, to ideally facilitate dynamic data transmission. However, more practically, operational and organizational problems are likely to occur during data transmission from the patient to the clinical setting, such as internet access, time zone, distinct data transfer protocols, and lack of data integration. Behavioral habits of consumers, as well as digital health literacy among key PGHD stakeholders, such as consumers and clinicians, are among the factors that participants identified, which can influence PGHD quality. The participant discussions further highlighted the need to create a patient-centered care model that includes defined technical standards, policies, rights, and responsibilities to ensure PGHD quality and make these data useful for clinical care.

### Convergence in Patient-Generated Health Data Stakeholders’ Expectations

The 4 groups of PGHD stakeholders in our workshop raised different expectations about each of the other stakeholders, whereas all participants reached consensus on the importance of promoting collaboration among stakeholders. Furthermore, participants emphasized the need for transparency on the redistribution of tasks and responsibilities given to consumers, as well as to the clinicians. Participants also stated that wearables should be designed in a way to reconcile the needs of both patients and clinicians. Another key discussion point focused on hearing the consumer’s voice in improving PGHD collection. Without this voice, it may result in the consumer’s lack of understanding on how to optimally contribute to PGHD collection and management processes, thus leading to poor quality data.

The scope of remote monitoring services is still limited to single programs for single diseases, and it has not yet broadened to multiple health conditions. Moreover, in current remote monitoring scenarios, PGHD flow mainly occurs among consumers, clinicians, and wearable manufacturers, without further actions entering EMR systems or without the involvement of other specialist clinical or nonclinical staff. Presently, health information professionals within clinical settings are not full participants in the PGHD process, primarily because of the lack of integrated infrastructure, which could optimally incorporate PGHD into the health care system. This particular gap points to the need to broaden the team of stakeholders involved in the process and better define tasks for ensuring PGHD quality across the flow.

Critically, there was consensus by the stakeholder groups that PGHD management should occur in a standardized way. Different types of PGHD could become part of clinical terminology standards, made possible through national efforts. For example, specific metadata could be standardized to codify patients as source data generators.

### Relation to Other Works

As stated, there is a paucity of research investigating PGHD quality for use in routine clinical practice [6,13,18]. This study included a wider range of PGHD stakeholders: consumers, clinicians, and wearable vendors, as well as health information professionals whose roles in PGHD management and quality assurance have not been investigated in previous research.

This is the first study of its kind, which brings various groups of PGHD stakeholders together to share their concerns and expectations with each other, with regard to ensuring PGHD quality. This process helped to reach a consensus among participants on clear responsibilities they could take to effectively collaborate for better patient care.

### Limitations and Future Work

The workshop participants were not representing an exhaustive range of PGHD stakeholders to explore quality issues from other perspectives. Further stages of this project will involve more groups of stakeholders. The workshop was focused on PGHD quality assurance for primary use of data, which falls within clinical care. Therefore, quality issues around secondary use of PGHD, such as utilization of PGHD in biomedical research, shared care plans, and mobile health surveillance, were not discussed.

In addition, the full spectrum of consumer and medical wearables was not represented among the participants. We purposely chose CGM devices as a medical wearable example, and we chose fitness trackers as a consumer wearable example in this workshop. CGM wearables are complex devices that require both automatic and manual data entry, calibration by using conventional glucometers, and a reliable connection to mobile apps and patient portals. Patients may also need to collect lifestyle data from consumer wearables, beside the CGM data, to provide a more comprehensive picture of their health status to their clinicians.

### Conclusions

This study used a participatory research method to identify (1) the problems of PGHD quality, (2) potential solutions to overcome the challenges, and (3) the PGHD stakeholders’ expectations toward PGHD quality assurance. Our approach to codevelop challenges, solutions, and expectations enabled us to engage various stakeholders to jointly share experience and concerns. Active and continuous collaboration among PGHD stakeholders, from wearable development to data production and use in patient care, is vital not only to ensure the quality of the data but also the quality of the consumer’s health care experience and clinical outcomes. The findings from this workshop will contribute to the development of practical recommendations toward PGHD quality assurance, which can be adopted by a wide range of PGHD stakeholders.

## Appendix

Multimedia Appendix 1 Workshop presentation slides.

## Abbreviations

CGM: continuous glucose monitoring
EMR: electronic medical record
PGHD: patient-generated health data
