# Supplementary material for: Quality Assurance of Health Wearables Data: Participatory Workshop on Barriers, Solutions, and Expectations
Source: JMIR Mhealth Uhealth. 2020 Jan 22;8(1):e15329. doi: 10.2196/15329 (PMC7003125; doi:10.2196/15329)
Supplement: Multimedia Appendix 1 [file mhealth_v8i1e15329_app1.pdf]

Multi-Stakeholder Workshop on

THE UNIVERSITY OF MELBOURNE

HaBIC Health & Biomedical Informatics Centre

Data Quality of Health Wearables

Is Wearable Data Reliable for Use in Clinical Care?

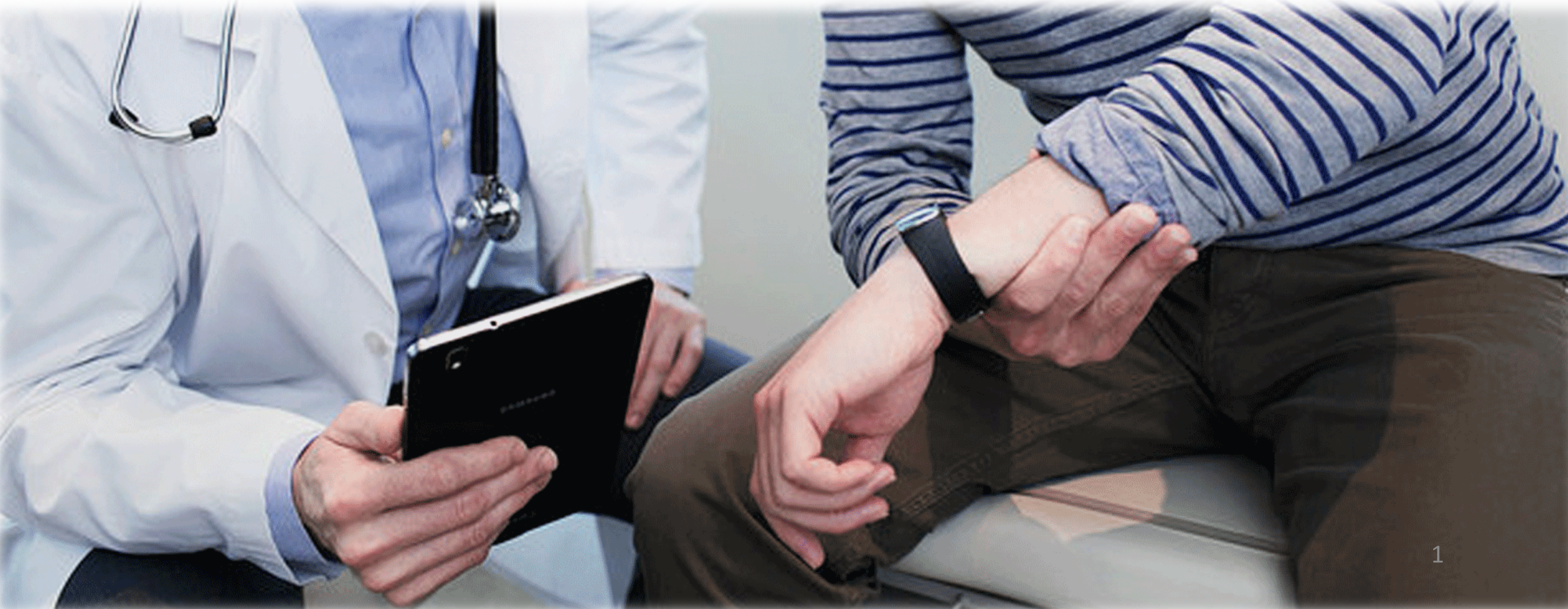

Agenda

- **Section 1: Welcome & Introduction**
- **Section 2: Presentation**
- **Section 3: Networking & Break**
- **Section 4: Group Discussion 1**
- **Section 5: Networking and Break**
- **Section 6: Group Discussion 2**
- **Section 7: Conclusion**

- Section 1: Welcome & Introduction
- **Section 2: Presentation**
- Section 3: Networking & Break
- Section 4: Group Discussion 1
- Section 5: Networking and Break
- Section 6: Group Discussion 2
- Section 7: Conclusion

REMOTE PATIENT MONITORING

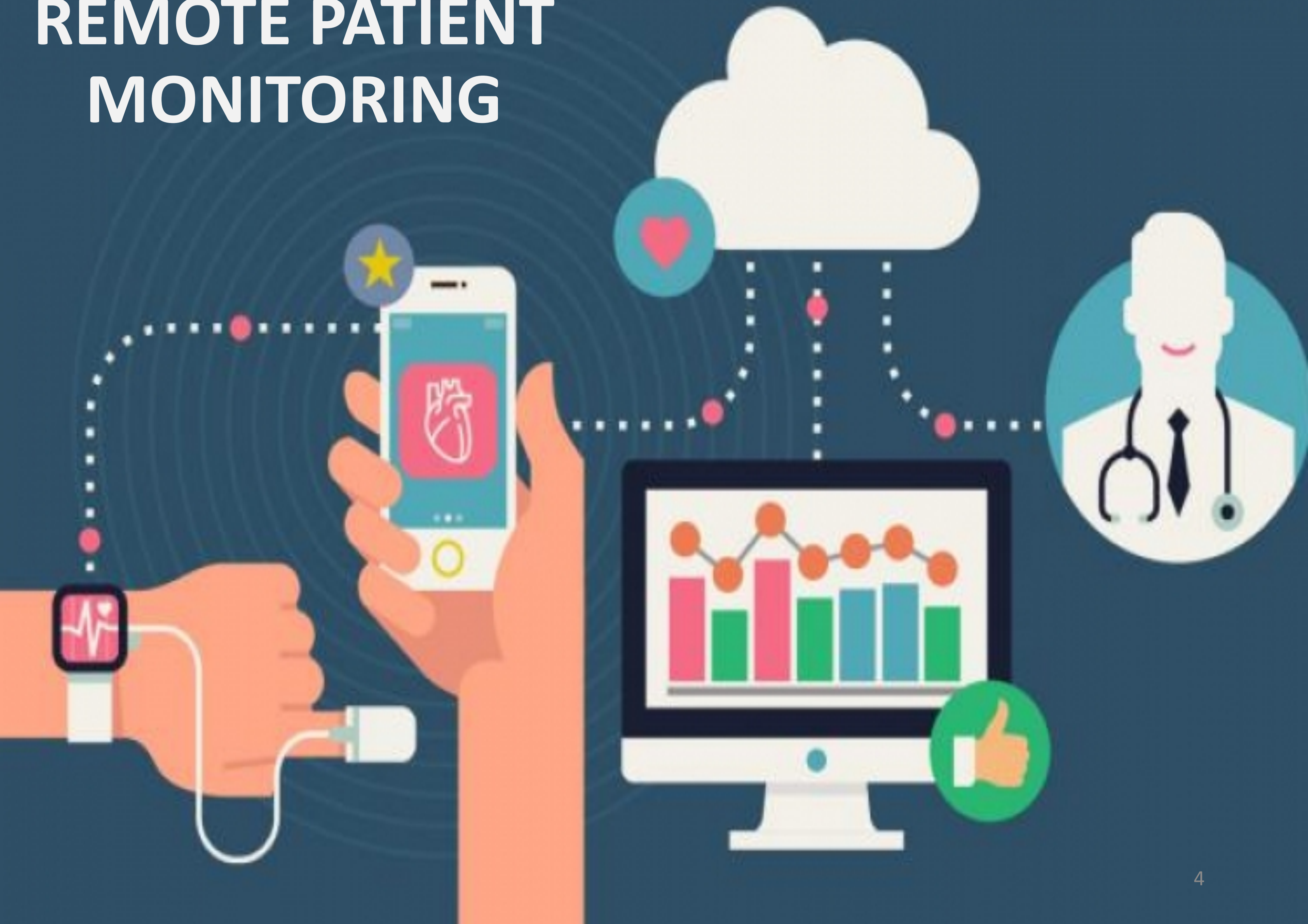

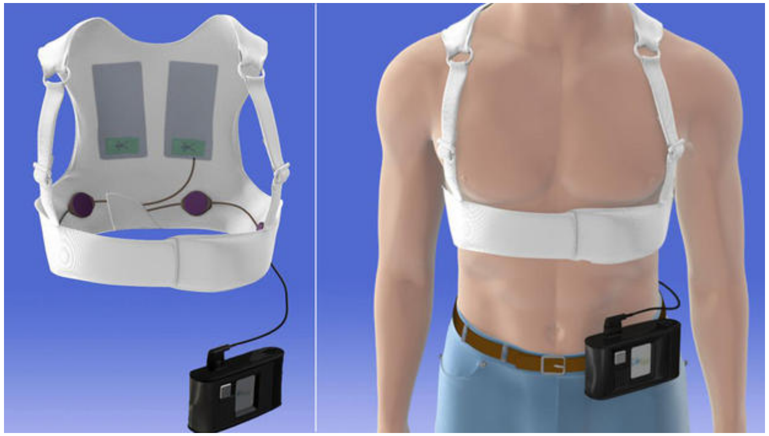

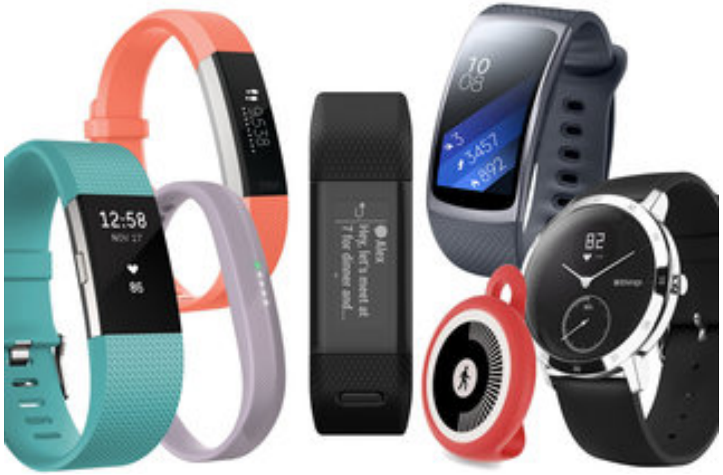

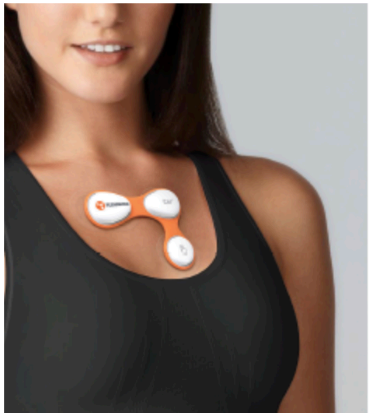

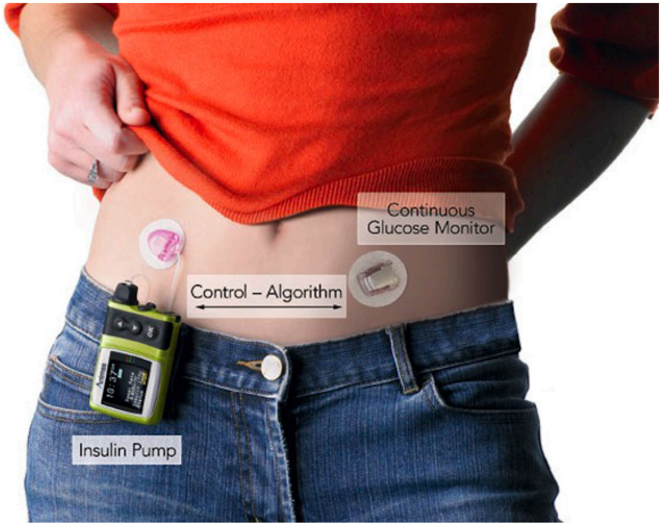
Continuous
Glucose Monitor
Control – Algorithm
Insulin Pump

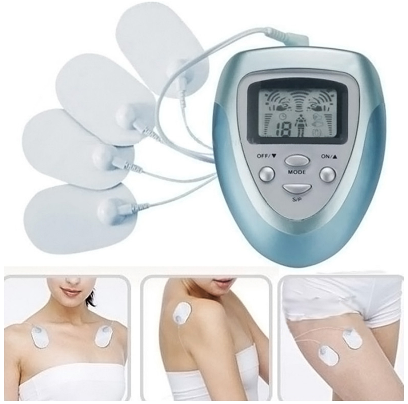

PATIENT GENERATED HEALTH DATA (PGHD)

- Patients, not clinicians, are primarily responsible for capturing or recording these data
- Data are collected outside the clinical setting
- Patients may choose how and with whom they can share their health data

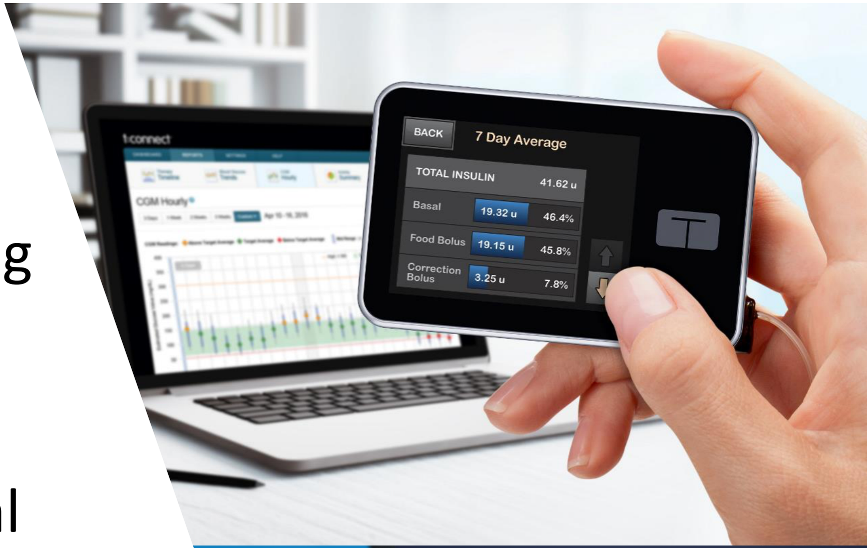

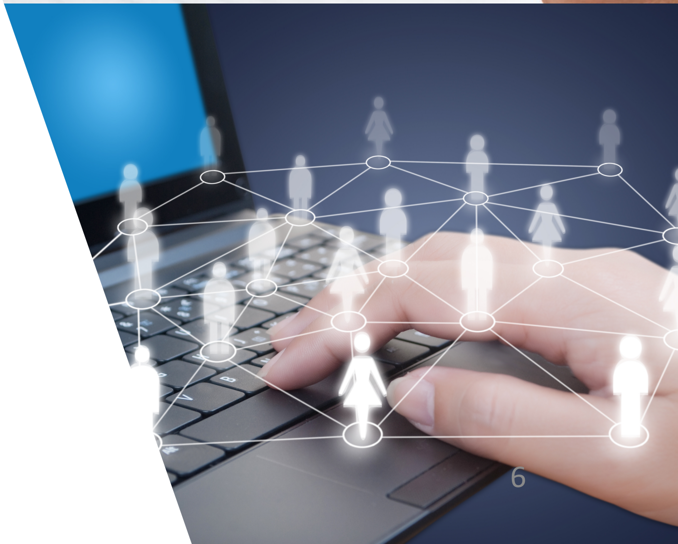

Why are we not using wearables data routinely in clinical care?

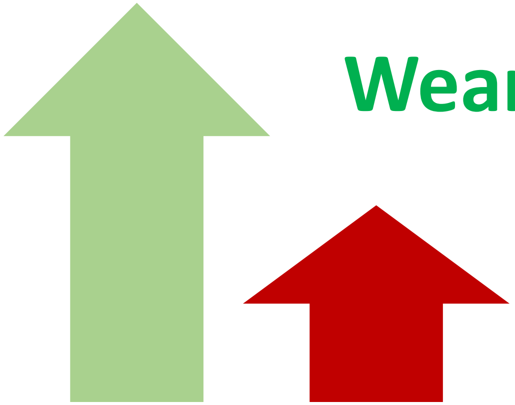

Wearable Devices Market

RPM and PGHD Adoption

Health consumers would use wearables and collect PGHD if recommended by the clinician

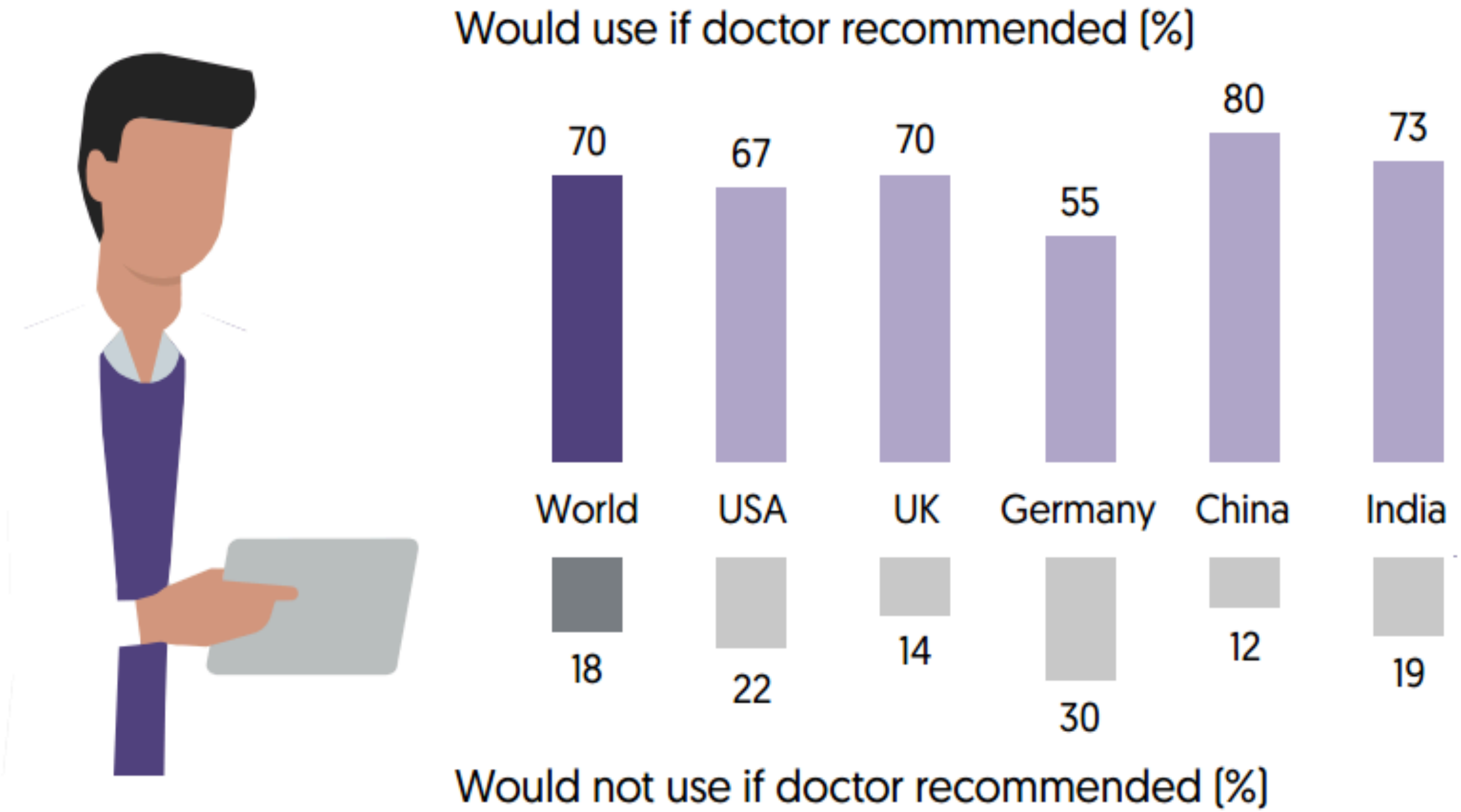

Source: Ipsos Global Connected Health Trends 2018
Base: 23,249 online adults aged 16-64 across 28 countries May 25 – June 8, 2018

Clinicians want wearables and PGHD in the care delivery continuum

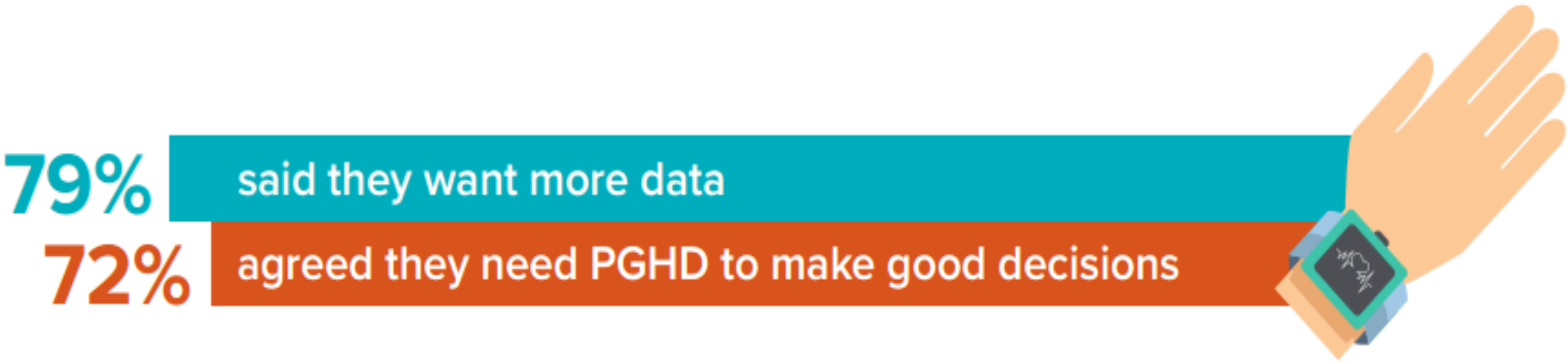

Source: HIMSS Media 2018
Base: 101 online responses from IT decision makers, business/administrative decision makers and healthcare professionals at hospitals and health systems.

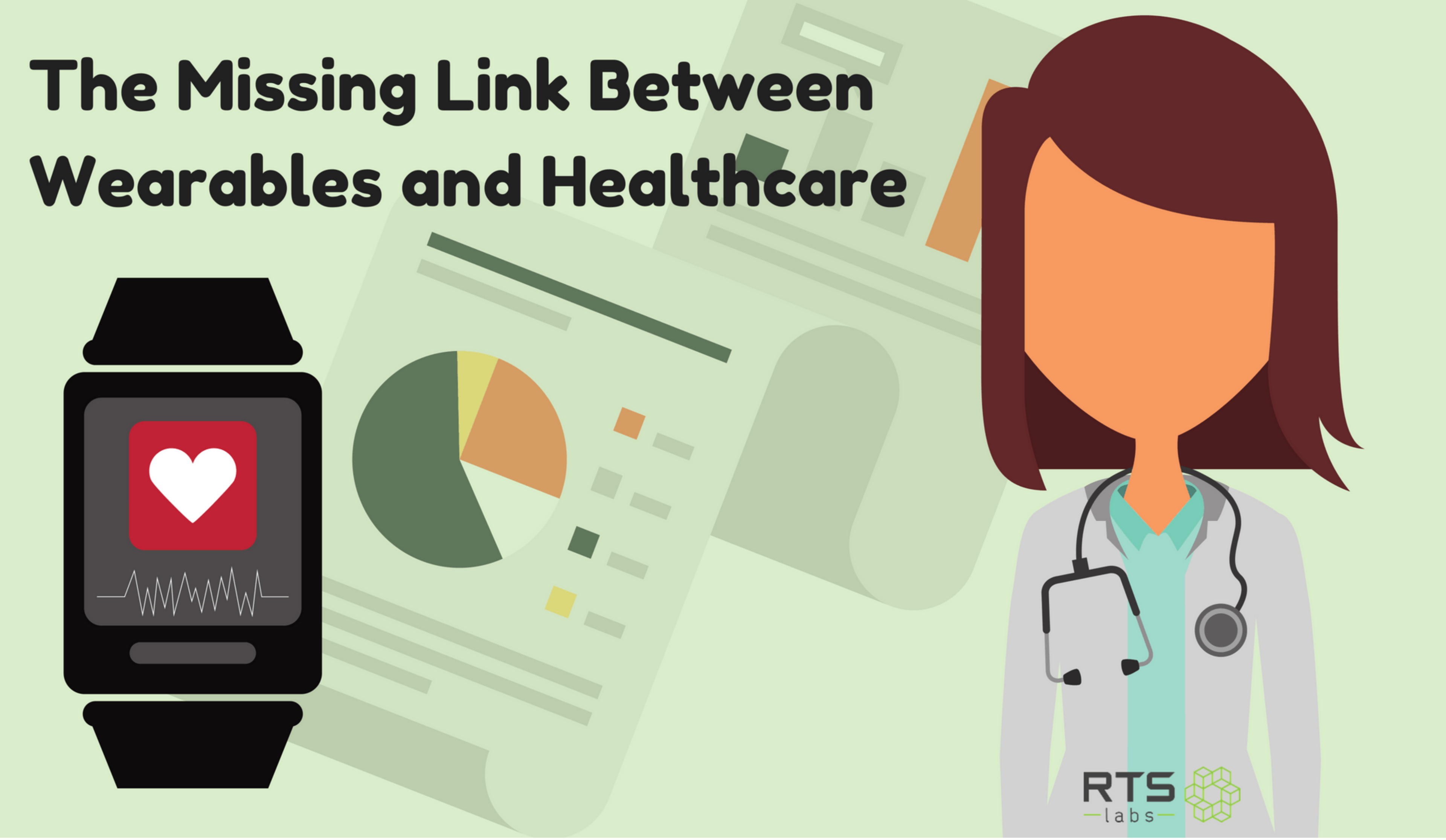

DATA QUALITY ASSURANCE

Who is responsible for for data quality assurance?

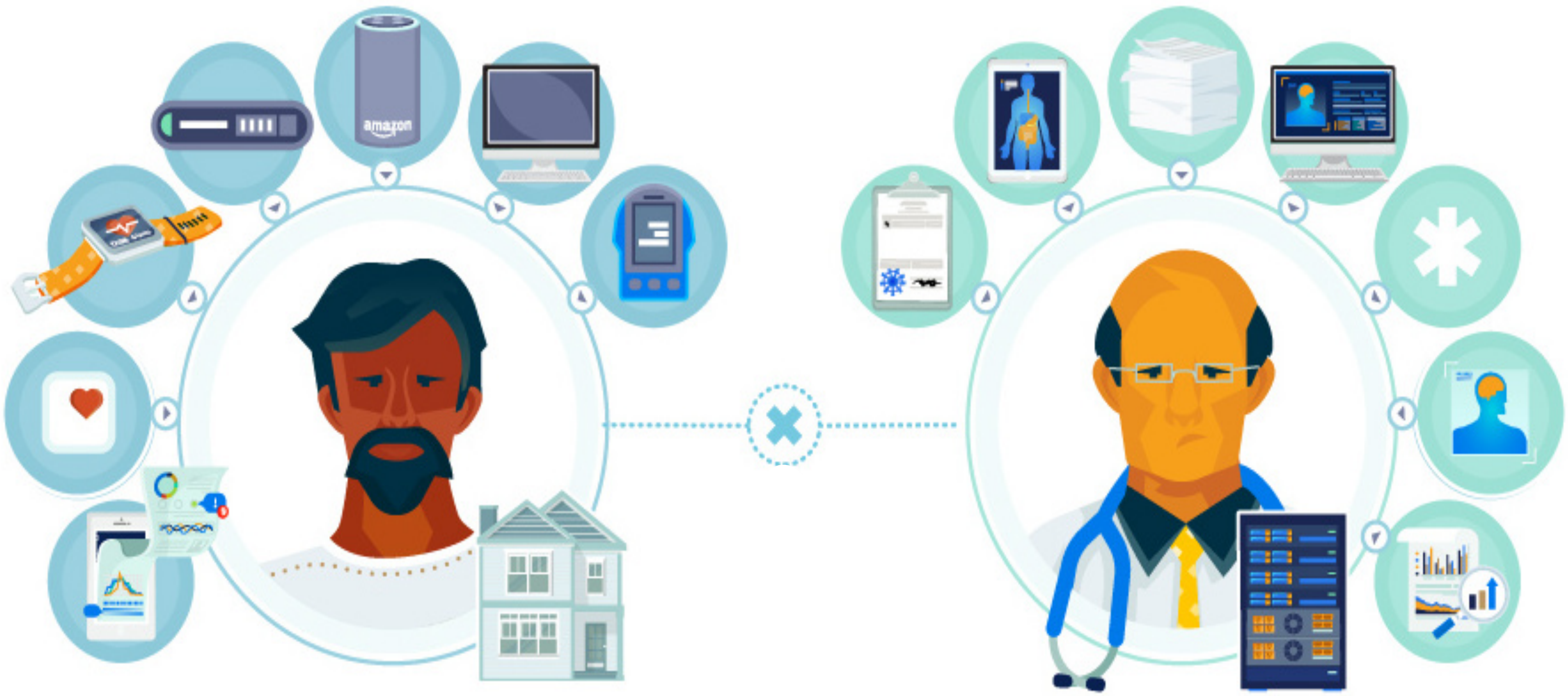

Our aim = data quality assurance for PGHD no matter the device, no matter the disease

Diabetes Care Volume 40, December 2017 1631

Check for updates

International Consensus on Use of Continuous Glucose Monitoring

Diabetes Care 2017;40:1631–1640 | https://doi.org/10.2337/dc17-1600

Thomas Danne,[1] Revital Nimri,[2] Tadej Battelino,[3] Richard M. Bergenstal,[4] Kelly L. Close,[5] J. Hans DeVries,[6] Satish Garg,[7] Lutz Heinemann,[8] Irl Hirsch,[9] Stephanie A. Amiel,[10] Roy Beck,[11] Emanuele Bosi,[12] Bruce Buckingham,[13] Claudio Cobelli,[14] Eyal Dassau,[15] Francis J. Doyle III,[15] Simon Heller,[16] Roman Hovorka,[17] Weiping Jia,[18] Tim Jones,[19] Olga Kordonouri,[1] Boris Kovatchev,[20] Aaron Kowalski,[21] Lori Laffel,[22] David Maahs,[13] Helen R. Murphy,[23] Kirsten Nørgaard,[24] Christopher G. Parkin,[25] Eric Renard,[26] Banshi Saboo,[27] Mauro Scharf,[28] William V. Tamborlane,[29] Stuart A. Weinzimer,[29] and Moshe Phillip[2]

Measurement of glycated hemoglobin (HbA_{1c}) has been the traditional method for assessing glycemic control. However, it does not reflect intra- and interday glycemic excursions that may lead to acute events (such as hypoglycemia) or postprandial hyperglycemia, which have been linked to both microvascular and macrovascular complications. Continuous glucose monitoring (CGM), either from real-time use (rtCGM) or intermittently viewed (iCGM), addresses many of the limitations inherent in HbA_{1c} testing and self-monitoring of blood glucose. Although both provide the means to move beyond the HbA_{1c} measurement as the sole marker of glycemic control, standardized metrics for analyzing CGM data are lacking. Moreover, clear criteria for matching people with diabetes to the most appropriate glucose monitoring methodologies, as well as standardized advice about how best to use the new information they provide, have yet to be established. In February 2017, the Advanced Technologies & Treatments for Diabetes (ATTD) Congress convened an international panel of physicians, researchers, and individuals with diabetes who are expert in CGM technologies to address these issues. This article summarizes the ATTD consensus recommendations and represents the current understanding of how CGM results can affect outcomes.

Glucose measurements are critical to effective diabetes management. Although measurement of glycated hemoglobin (HbA_{1c}) has been the traditional method for assessing glycemic control, it does not reflect intra- and interday glycemic excursions that may lead to acute events (such as hypoglycemia) or postprandial hyperglycemia, which have been linked to both microvascular and macrovascular complications. Moreover, although self-monitoring of blood glucose (SMBG) has been shown to improve glyce-

[1]*Diabetes Centre for Children and Adolescents, Children's and Youth Hospital "Auf Der Bult," Hannover, Germany*
[2]*The Myrtle and Henry Hirsch National Center for Childhood Diabetes, The Jesse and Sara Lea Shafer Institute of Endocrinology and Diabetes, Schneider Children's Medical Center of Israel, Petah Tikva, Israel*
[3]*Department of Pediatric Endocrinology, Diabetes and Metabolic Diseases, University Children's Hospital, Ljubljana University Medical Centre, and Faculty of Medicine, University of Ljubljana, Ljubljana, Slovenia*
[4]*International Diabetes Center at Park Nicollet, Minneapolis, MN*
[5]*Close Concerns, San Francisco, CA*
[6]*Academic Medical Center, University of Amsterdam, Amsterdam, the Netherlands*
[7]*University of Colorado Denver and Barbara Davis Center for Diabetes, Aurora, CO*
[8]*Science & Co, Düsseldorf, Germany*
[9]*Division of Metabolism, Endocrinology and* [illegible]

Word

HRS Expert Consensus Statement on remote interrogation and monitoring for cardiovascular implantable electronic devices

CrossMark

David Slotwiner, MD, FHRS, FACC (Chair),[1,#] Niraj Varma, MD, PhD, FRCP (Co-chair),[2,#] Joseph G. Akar, MD, PhD,[3] George Annas, JD, MPH,[4] Marianne Beardsall, MN/NP, CCDS, FHRS,[5] Richard I. Fogel, MD, FHRS,[6] Nestor O. Galizio, MD,[7*] Taya V. Glotzer, MD, FHRS, FACC,[8] Robin A. Leahy, RN, BSN, CCDS, FHRS,[9] Charles J. Love, MD, CCDS, FHRS, FACC, FAHA,[10] Rhondalyn C. McLean, MD,[11†] Suneet Mittal, MD, FHRS,[12] Loredana Morichelli, RN, MSN,[13] Kristen K. Patton, MD,[14‡] Merritt H. Raitt, MD, FHRS,[15] Renato Pietro Ricci, MD,[13§] John Rickard, MD, MPH,[16] Mark H. Schoenfeld, MD, CCDS, FHRS, FACC, FAHA,[17] Gerald A. Serwer, MD, FHRS, FACC,[18||] Julie Shea, MS, RNCS, FHRS, CCDS,[19] Paul Varosy, MD, FHRS, FACC, FAHA,[20] Atul Verma, MD, FHRS, FRCPC,[5] Cheuk-Man Yu, MD, FACC, FRCP, FRACP[21¶]

From the [1]*Hofstra School of Medicine, North Shore - Long Island Jewish Health System, New Hyde Park, New York,* [2]*Cleveland Clinic, Cleveland, Ohio,* [3]*Yale University School of Medicine, New Haven, Connecticut,* [4]*Boston University School of Public Health, Boston, Massachusetts,* [5]*Southlake Regional Health Centre, Newmarket, Ontario, Canada,* [6]*St. Vincent Medical Group, Indianapolis, Indiana,* [7]*Favaloro Foundation University Hospital, Buenos Aires, Argentina,* [8]*Hackensack University Medical Center, Hackensack, New Jersey,* [9]*Sanger Heart & Vascular Institute, Carolinas HealthCare System, Charlotte, North Carolina,* [10]*New York University Langone Medical Center, New York City, New York,* [11]*University of Pennsylvania Health System, Philadelphia, Pennsylvania,* [12]*The Arrhythmia Institute at Valley Hospital, New York, New York,* [13]*Department of Cardiovascular Diseases, San Filippo Neri Hospital, Rome, Italy,* [14]*University of Washington, Seattle, Washington,* [15]*VA Portland Health Care System, Oregon Health & Science University, Knight Cardiovascular Institute, Portland, Oregon,* [16]*Johns Hopkins University, Baltimore, Maryland,* [17]*Yale University School of Medicine, Yale-New Haven Hospital Saint Raphael Campus, New Haven, Connecticut,* [18]*University of Michigan Congenital Heart Center, University of Michigan Health Center, Ann Arbor, Michigan,* [19]*Brigham and Women's Hospital, Boston, Massachusetts,* [20]*Veterans Affairs Eastern Colorado Health Care System, University of Colorado, Denver, Colorado, and* [21]*Department of Medicine and Therapeutics, Prince of Wales Hospital, The Chinese University of Hong Kong, Hong Kong, China.*

#These authors contributed equally to this work. *Representative for the Latin American Society of Cardiac Pacing and Electrophysiology (*Sociedad Latinoamericana de Estimulación Cardíaca y Electrofisiología* [SOLAECE]); †Representative for the American Heart Association (AHA);

TABLE OF CONTENTS

Introduction e70
Remote Interrogation vs Remote Monitoring e70

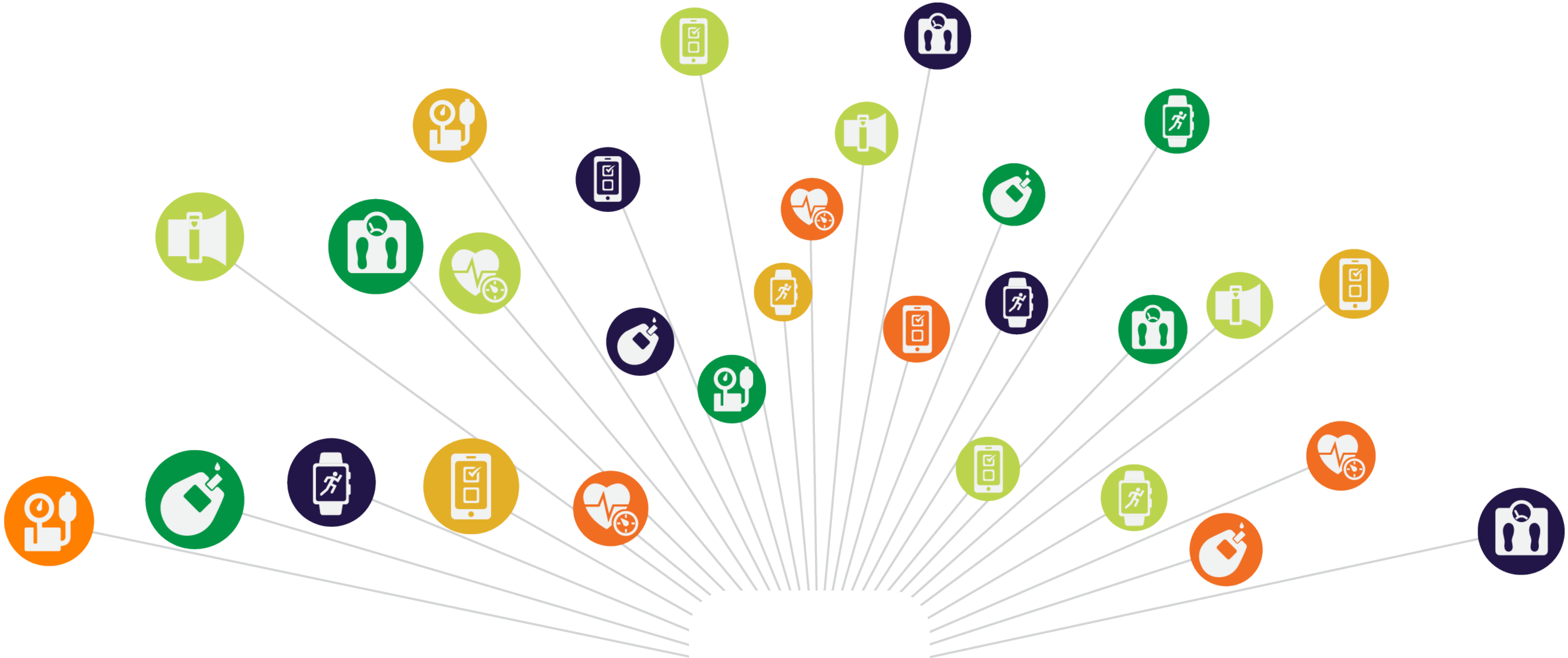

PGHD

Is it understandable?

Is it accurate?

Is it relevant?

Is it consistent with other data?

Is it accessible?

Is it complete?

Is it available at anytime?

Is it compliant with clinical standards?

Why Should We Care about Data Quality?

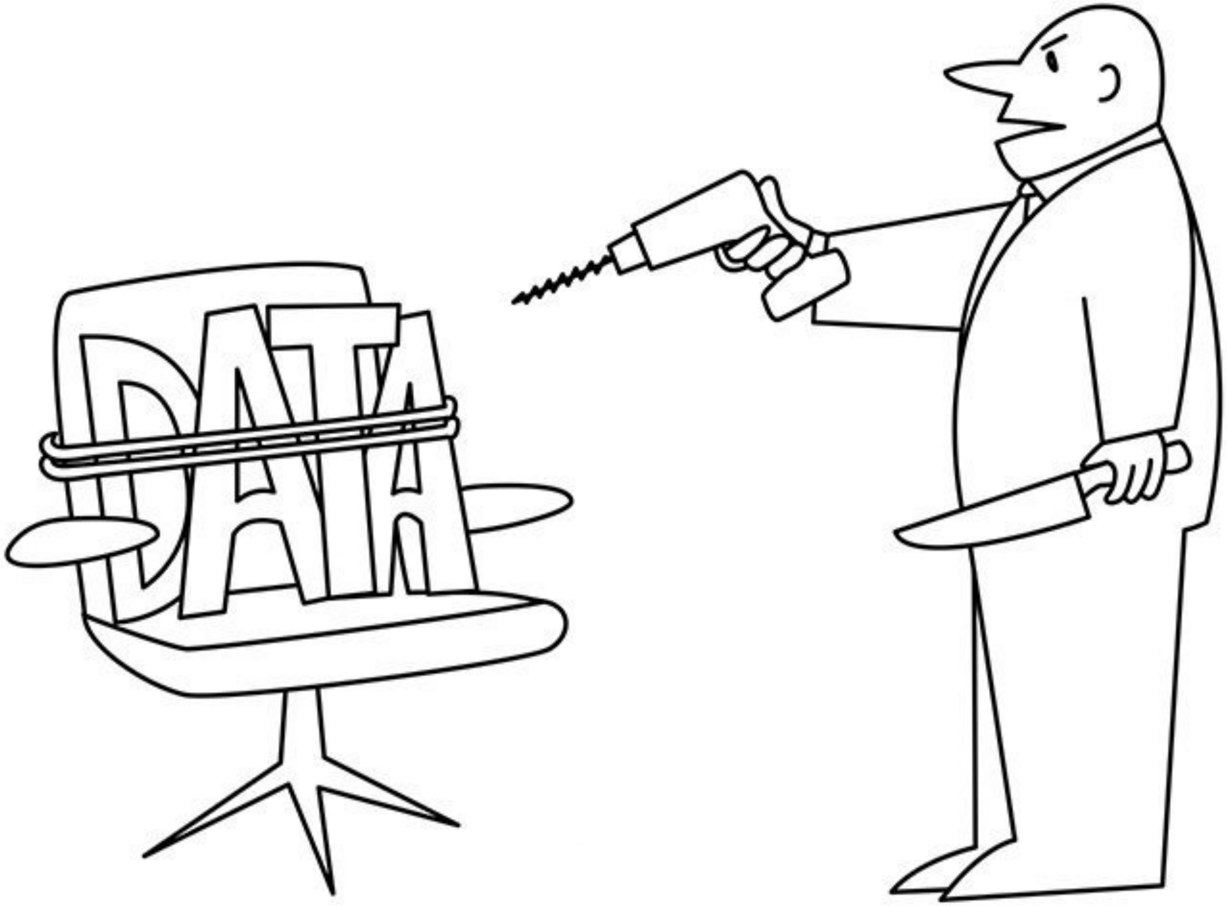

"If you don't reveal some insights soon, I'm going to be forced to slice, dice, and drill!"

This is Steve,
35 years old, diabetic type 1

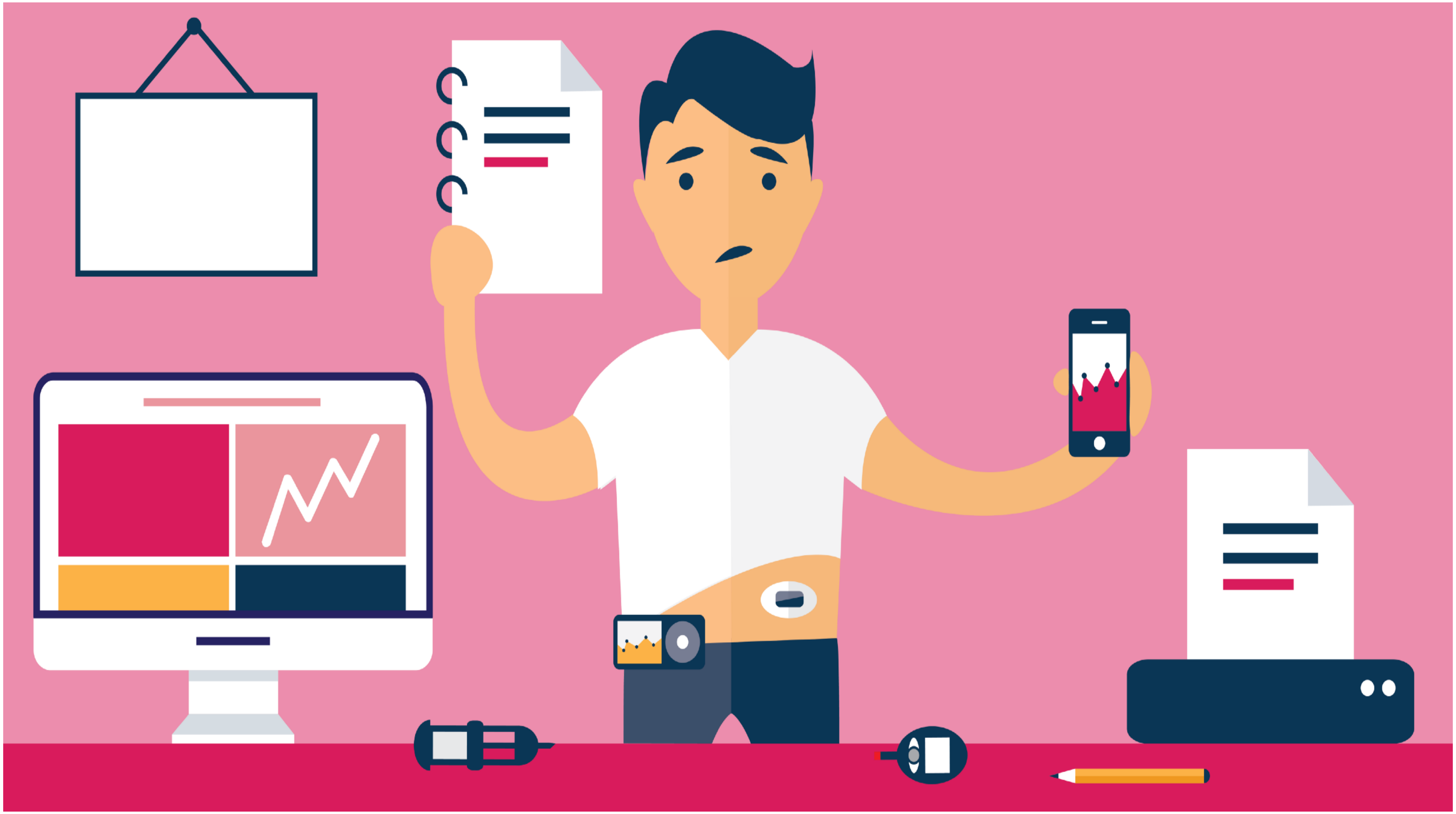

1 Data Accessibility

“authorised users (and no one else) can access data”

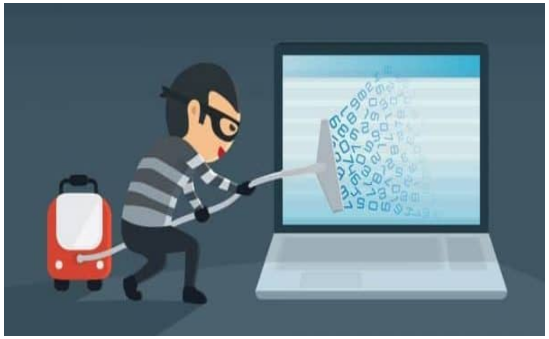

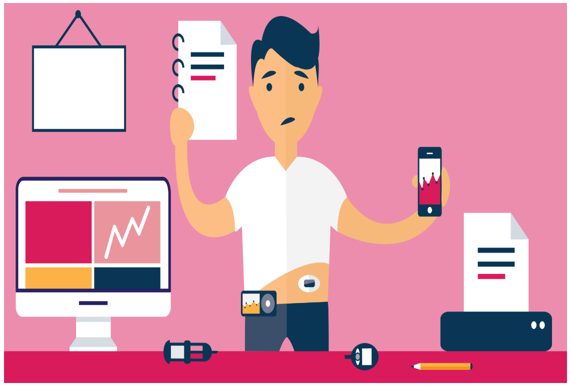

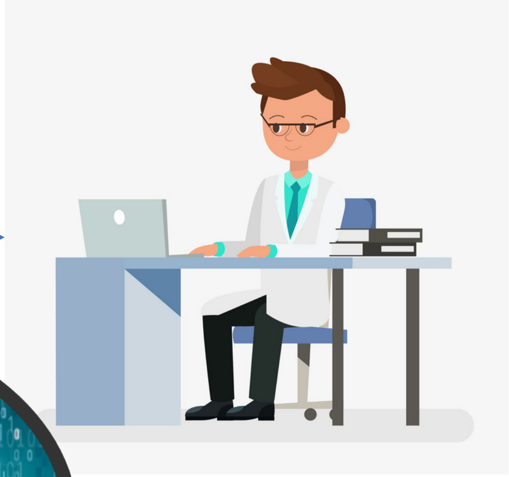

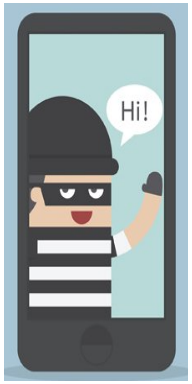

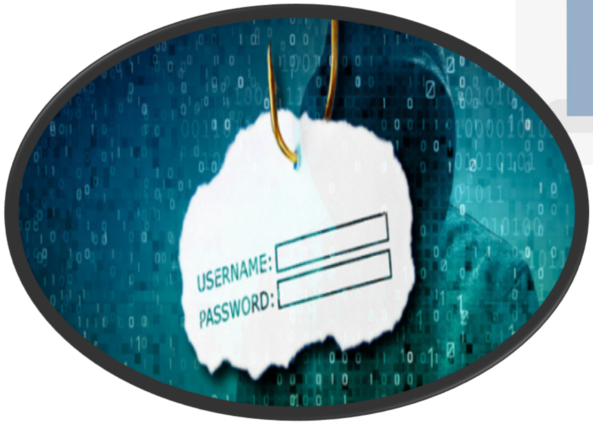

2 Data Accuracy

“Data are free from errors”

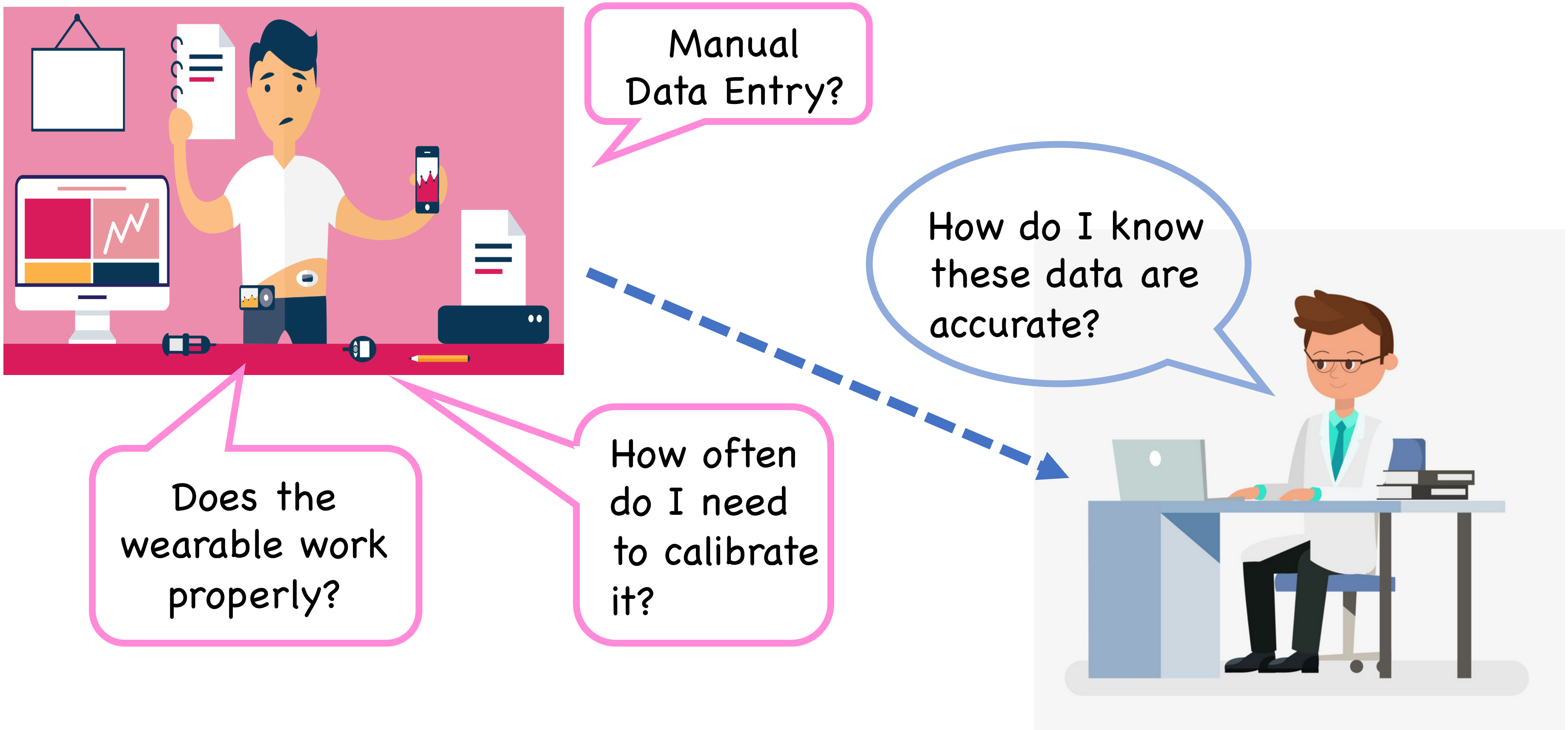

3 Data Completeness

“There are no data missing”

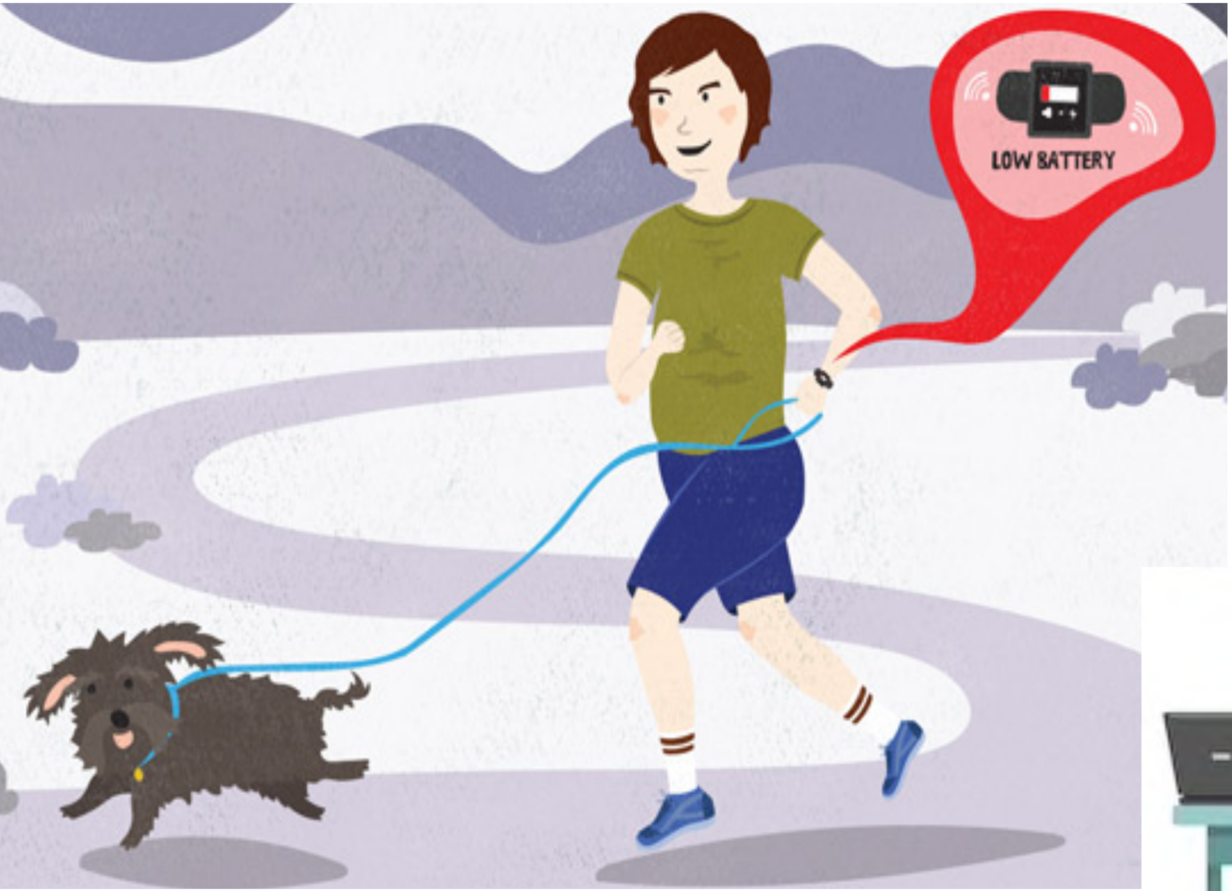

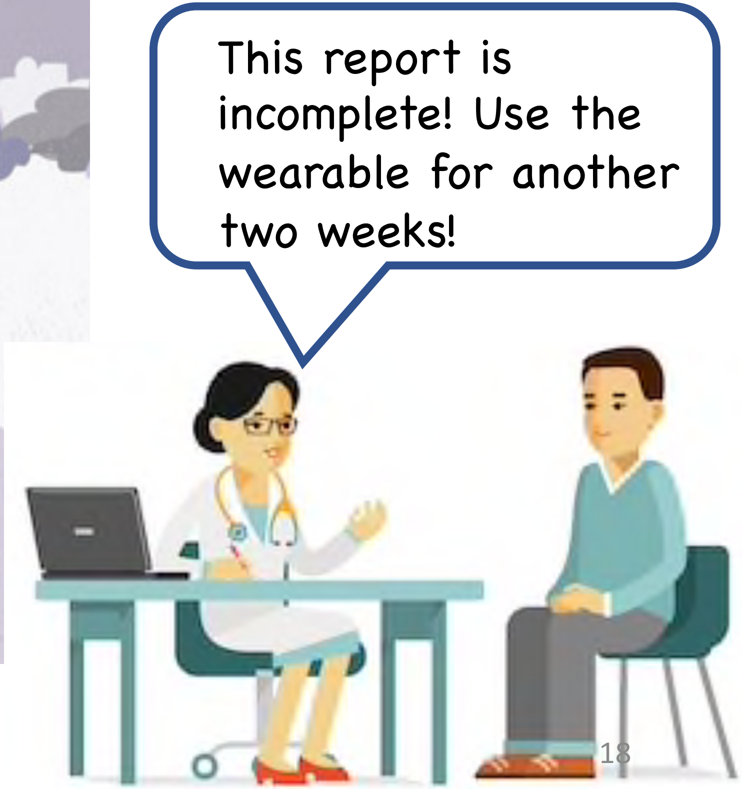

4 Data Consistency

“Data from different devices, convey the same meaning”

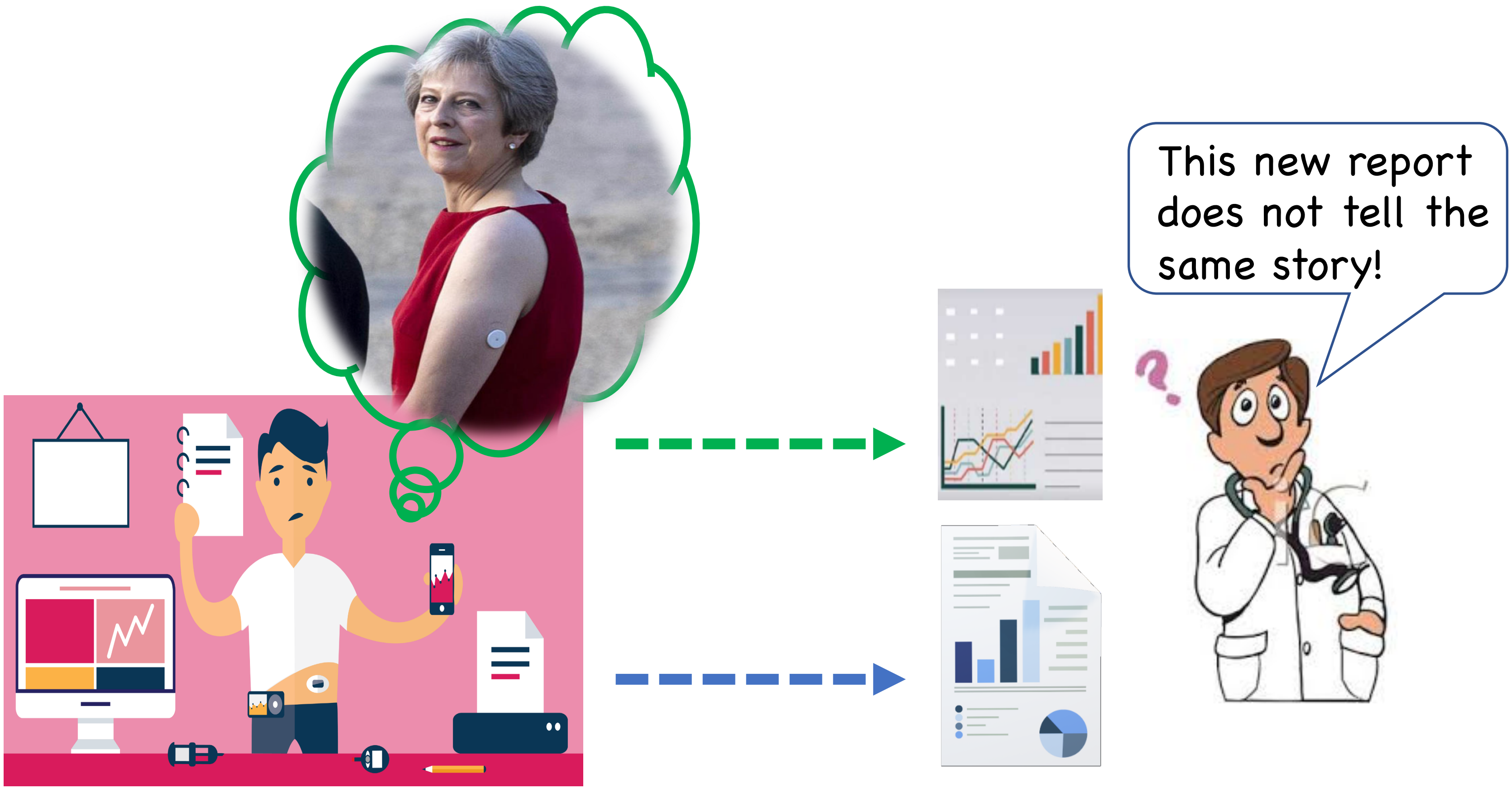

5 Data Interpretability

“The data presentation highlights the key message”

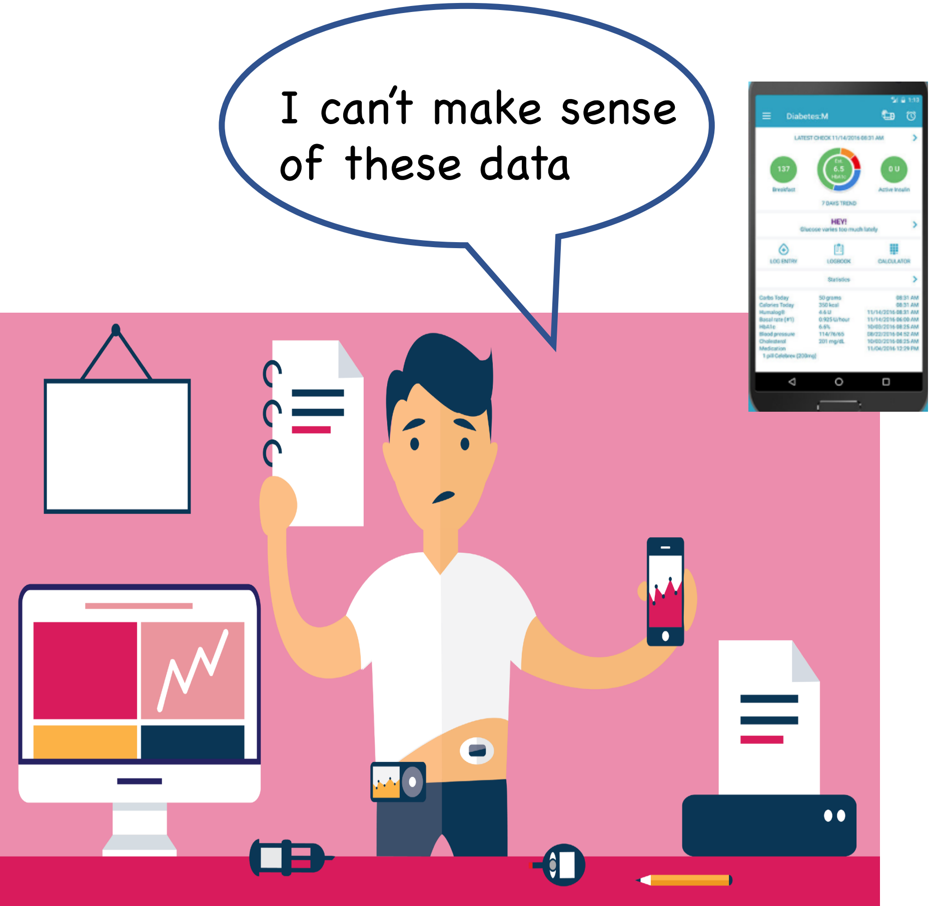

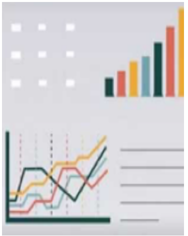

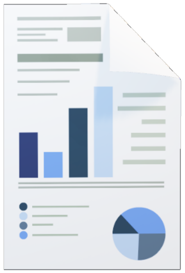

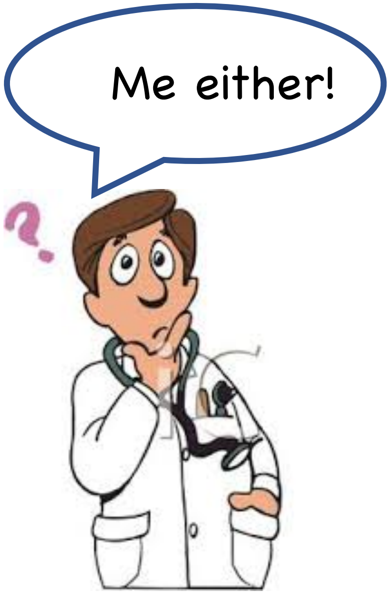

6 Data Relevancy

"The data being collected are pertinent to the standard of care"

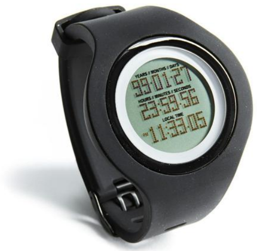

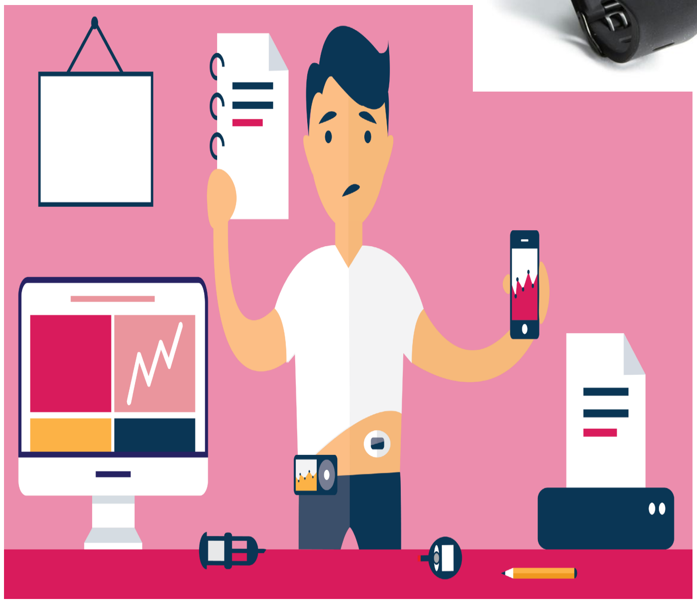

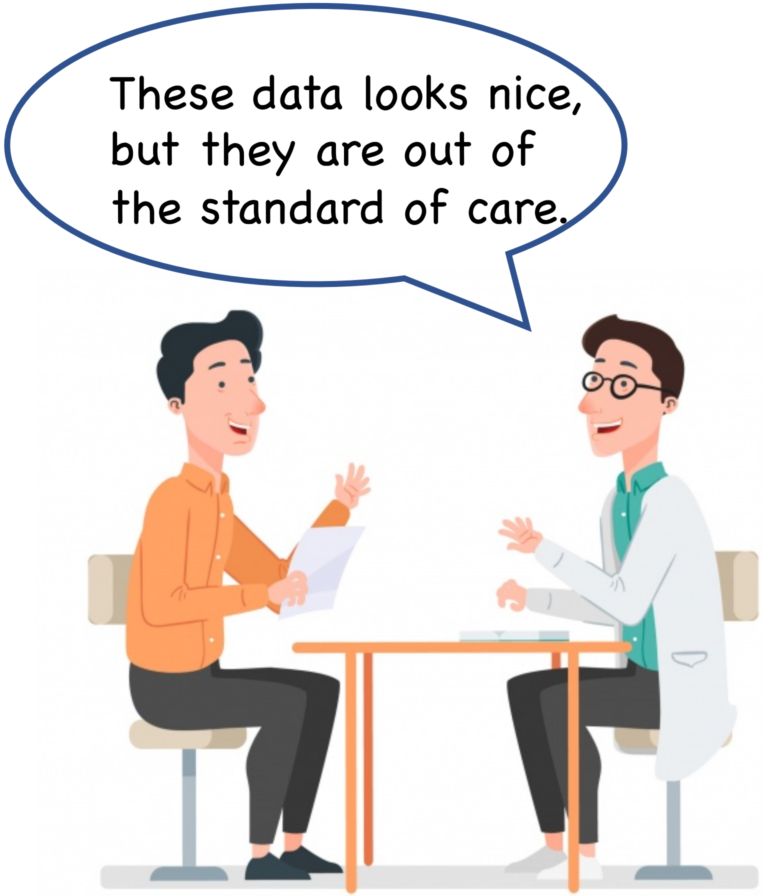

7 Data Timeliness

“Up-to-date data are available when needed”

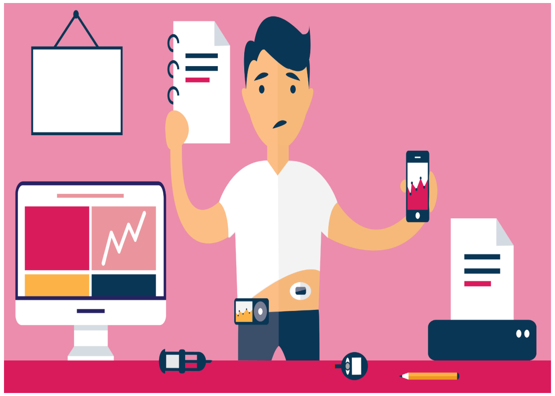

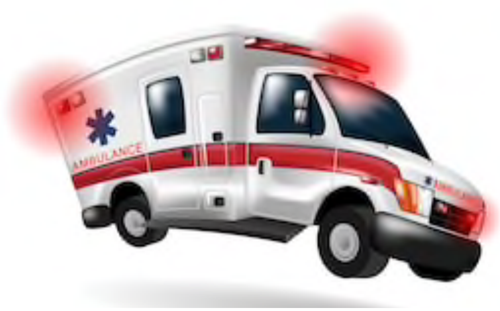

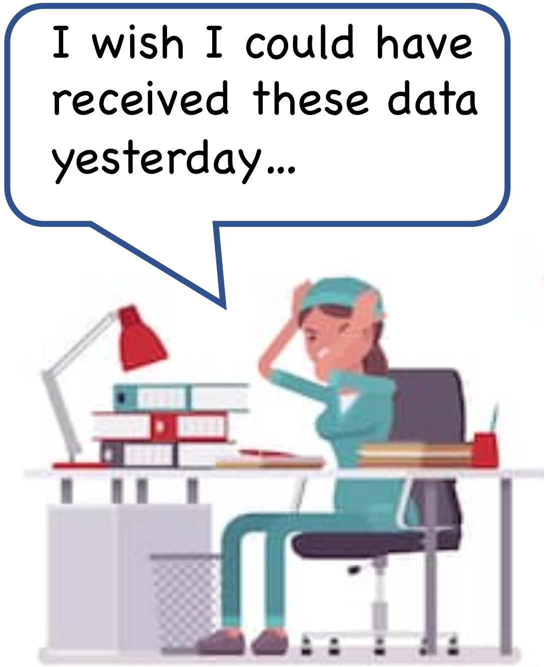

- Section 1: Introduction
- Section 2: Presentation
- **Section 3: Networking & Break**
- Section 4: Group Discussion 1
- Section 5: Networking and Break
- Section 6: Group Discussion 2
- Section 7: Conclusion

- Section 1: Introduction
- Section 2: Presentation
- Section 3: Networking & Break
- **Section 4: Group Discussion 1**
- Section 5: Networking and Break
- Section 6: Group Discussion 2
- Section 7: Conclusion

PGHD Quality in Remote Patient Monitoring
Problems and Ideas

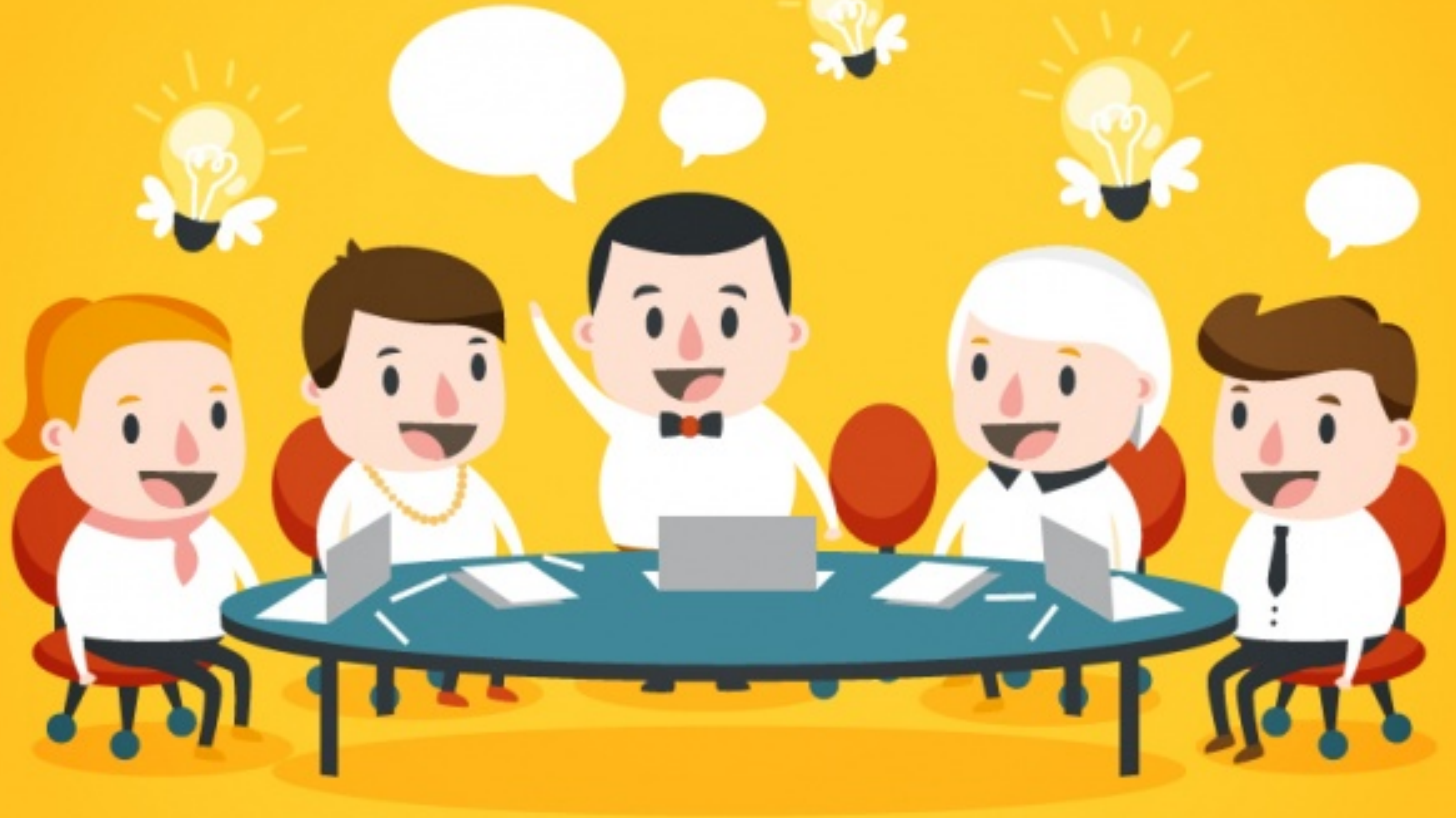

- Section 1: Introduction
- Section 2: Presentation
- Section 3: Networking & Break
- Section 4: Group Discussion 1
- **Section 5: Networking and Break**
- Section 6: Group Discussion 2
- Section 7: Conclusion

- Section 1: Introduction
- Section 2: Presentation
- Section 3: Networking & Break
- Section 4: Group Discussion 1
- Section 5: Networking and Break
- **Section 6: Group Discussion 2**
- Section 7: Conclusion

Stakeholders’ Expectations about PGHD Quality Assurance

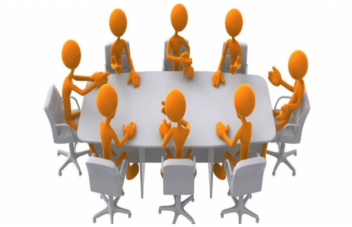

Health Consumers Group

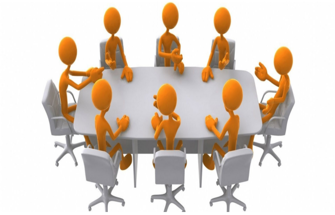

Healthcare Providers Group

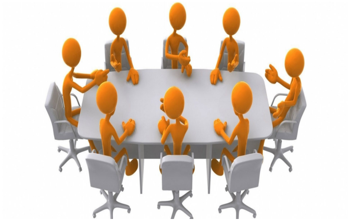

Health Information Professionals Group

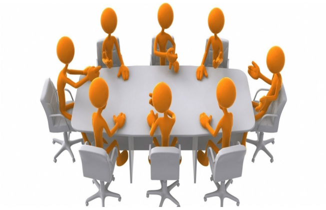

Wearable Vendors/Data Integration Service Providers Group

- Section 1: Introduction
- Section 2: Presentation
- Section 3: Networking & Break
- Section 4: Group Discussion 1
- Section 5: Networking and Break
- Section 6: Group Discussion 2
- **Section 7: Conclusion**

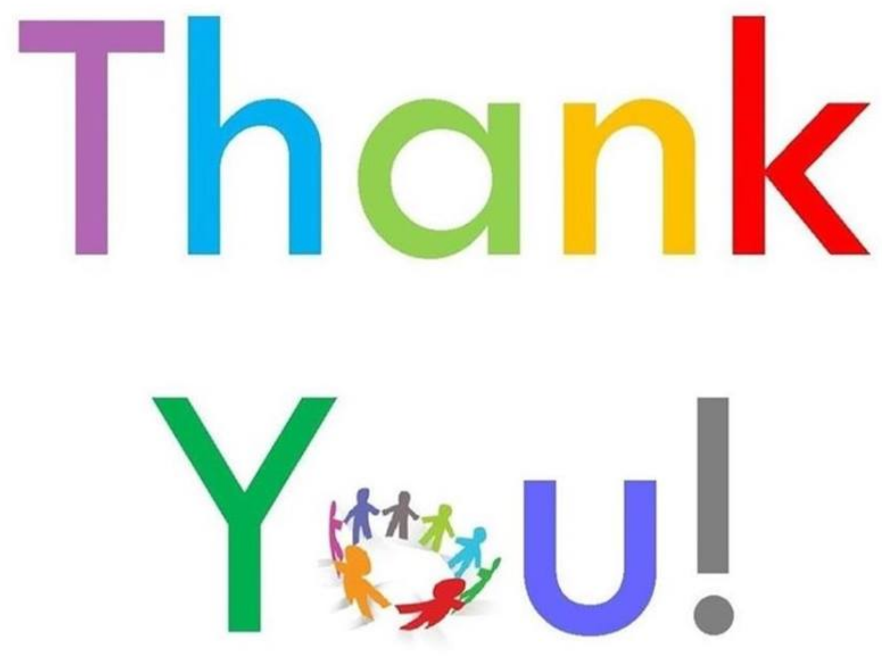

rabdolkhani@student.unimelb.edu.au

Google Scholar, LinkedIn: Robab Abdolkhani

Twitter: @HABIC_UOM
